# Anti-metabolic agent pegaspargase plus PD-1 antibody sintilimab for first-line treatment in advanced natural killer T cell lymphoma

**DOI:** 10.1038/s41392-024-01782-8

**Published:** 2024-03-06

**Authors:** Jie Xiong, Shu Cheng, Xiao Gao, Shan-He Yu, Yu-Ting Dai, Xin-Yun Huang, Hui-Juan Zhong, Chao-Fu Wang, Hong-Mei Yi, Hao Zhang, Wei-Guo Cao, Rong Li, Wei Tang, Yan Zhao, Peng-Peng Xu, Li Wang, Wei-Li Zhao

**Affiliations:** 1 Shanghai Institute of Hematology, State Key Laboratory of Medical Genomics, National Research Center for Translational Medicine at Shanghai, Ruijin Hospital Affiliated to Shanghai Jiao Tong University School of Medicine, Shanghai, China; 2 Department of Nuclear Medicine, Shanghai Ruijin Hospital, Shanghai Jiao Tong University School of Medicine, Shanghai, China; 3 Department of Pathology, Shanghai Ruijin Hospital, Shanghai Jiao Tong University School of Medicine, Shanghai, China; 4 Department of Otolaryngology, Shanghai Ruijin Hospital, Shanghai Jiao Tong University School of Medicine, Shanghai, China; 5 Department of Radiation, Shanghai Ruijin Hospital, Shanghai Jiao Tong University School of Medicine, Shanghai, China; 6 Department of Hematology, Navy Medical Center of PLA, Shanghai, China; 7 Pôle de Recherches Sino-Français en Science du Vivant et Génomique, Laboratory of Molecular Pathology, Shanghai, China

**Keywords:** Haematological cancer, Clinical trials, Immunotherapy

## Abstract

Natural killer T cell lymphoma (NKTCL) is highly aggressive, with advanced stage patients poorly responding to intensive chemotherapy. To explore effective and safe treatment for newly diagnosed advanced stage NKTCL, we conducted a phase II study of anti-metabolic agent pegaspargase plus PD-1 antibody sintilimab (NCT04096690). Twenty-two patients with a median age of 51 years (range, 24–74) were enrolled and treated with induction treatment of pegaspargase 2500 IU/m^2^ intramuscularly on day 1 and sintilimab 200 mg intravenously on day 2 for 6 cycles of 21 days, followed by maintenance treatment of sintilimab 200 mg for 28 cycles of 21 days. The complete response and overall response rate after induction treatment were 59% (95%CI, 43–79%) and 68% (95%CI, 47–84%), respectively. With a median follow-up of 30 months, the 2 year progression-free and overall survival rates were 68% (95%CI, 45–83%) and 86% (95%CI, 63–95%), respectively. The most frequently grade 3/4 adverse events were neutropenia (32%, *n* = 7) and hypofibrinogenemia (18%, *n* = 4), which were manageable and led to no discontinuation of treatment. Tumor proportion score of PD-L1, peripheral blood high-density lipoprotein cholesterol, and apolipoprotein A-I correlated with good response, while PD-1 on tumor infiltrating lymphocytes and peripheral Treg cells with poor response to pegaspargase plus sintilimab treatment. In conclusion, the chemo-free regimen pegaspargase plus sintilimab was effective and safe in newly diagnosed, advanced stage NKTCL. Dysregulated lipid profile and immunosuppressive signature contributed to treatment resistance, providing an alternative therapeutic approach dual targeting fatty acid metabolism and CTLA-4 in NKTCL.

## Introduction

Natural killer T cell lymphoma (NKTCL) is a CD56 + /cytoCD3+ lymphoma subtype, which is highly aggressive and caused by persistent infection of Epstein-Barr virus (EBV).^1^ It comprises 31 and 8% of mature T/NK-cell lymphoma in Asian and European/US countries, respectively, according to a prospective cohort study of T-cell project.^2^ Aberrant glutamine metabolism is involved in the pathogenesis of NKTCL.^3^ Anti-metabolic agent asparaginase exerts an anti-tumor effect by depleting extracellular asparagine and inhibiting glutamine-dependent tumor cell growth.^4^ Indeed, asparaginase-containing chemotherapy in combination with radiotherapy achieves high efficacy in early stage NKTCL (includes patients with Ann Arbor stage I and II).^5–7^ However, advanced stage NKTCL (includes patients with Ann Arbor stage III and IV), accounting for approximately 30% of the patients, presents an inferior prognosis with a median survival of 4–7 months.^8^ First-line intensive chemotherapy like SMILE regimen (dexamethasone, methotrexate, ifosfamide, asparaginase, and etoposide) has a complete response (CR) rate of 40% (8/20) in advanced stage NKTCL, which should be safely administrated with careful attention to adverse effects (AEs).^9^ DDGP regimen (dexamethasone, cisplatin, gemcitabine, and pegaspargase) shows better tolerability with 1 year progression-free survival (PFS) as 86% and 2 year overall survival (OS) as 74%.^10^ However, an effective and safe chemo-free regimen has not yet been attempted to treat advanced stage NKTCL.

We previously identified three genetic alteration-based molecular subtypes, namely TSIM subtype with *TP53* mutation, JAK-STAT mutation/amplification, del6q21, and amp9p24.1/PD-L1/2, MB subtype with *MGA* mutation and LOH at the *BRDT* locus, as well as HEA subtype with *HDAC9*, *EP300*, and *ARID1A* mutation, informing molecular networks of NKTCL pathgenesis.^11^ Beside, EBV gene expression patterns were associated with molecular subtypes.^11^ As a mechanism of action, EBV modulates immune-related oncogenic signaling and reprograms tumor microenvironment via GPCR interactome in NKTCL.^12^ Alternatively, EBV interferes with the expression of immune checkpoints, notably programmed death 1 (PD-1) and programmed death ligand 1 (PD-L1), found to be overexpressed in EBV-associated nasopharyngeal and gastric adenocarcinomas.^13^ In NKTCL, increased expression of tumoral PD-L1 is also induced by EBV latent membrane protein 1 (LMP1)^14^ and related to oncogenic activation of STAT3 pathway.^15^ Clinically, PD-1 blockade achieves a high CR rate in NKTCL failing to asparaginase-based regimens and tumoral PD-L1 expression correlates with favorable outcomes of the patients.^16,17^ More recently, our pre-clinical study has shown that asparaginase therapeutically targets glutamine addiction and sensitizes PD-1 antibody by inducing tumor PD-L1 expression and modulating cytotoxic T cell activity,^3^ providing the clinical rationale for co-targeting metabolic vulnerability and immune checkpoints in treating NKTCL.

Although clinical studies report promising efficacy of PD-1 antibody in treating multiple cancers, such as lymphoma, melanoma, lung cancer, gastric cancer, breast cancer, etc, not all patients respond to treatment, encouraging further investigation on the therapeutic mechanisms and prognostic biomarkers. Tumor infiltrating lymphocytes (TIL) and tumoral PD-1 expression are potential predictors,^18^ but their efficacy in NKTCL remains to be clarified. Besides, clonal expansion of T cells upon treatment indicates therapeutic response to PD-1 antibody.^19^ As defined with single-cell transcriptome, immune signatures including *PD-L1*^+^ immunoregulatory dendritic cells (DCs), *CCR2*^+^ or *MMP9*^+^ macrophages, and major histocompatibility complex class I/II expressing cancer cell are positively correlated with T cell expansion.^19^ Meanwhile, immune signatures including *TCF7*^+^/*GZMK*^+^ undifferentiated pre-effector/memory T cells and *CX3CR1*^+^/*C3*^+^ inhibitory macrophages are negatively correlated with T cell expansion.^19^ In clinical settings, however, either tumor biopsies after treatment or single-cell transcriptome technology are not easily accessible. Recent studies showed that circulating immune cell signatures, such as CD4^+^ T cell TCR repertoire diversity, natural killer (NK) cell abundance, and newly identified CD3^-^CD68^+^CD4^+^GrB^+^ cell subset, correlating with therapeutic response to PD-1 blockade in classical Hodgkin’s lymphoma,^20^ inspiring us to assess peripheral blood biomarkers relating to treatment response.

In the present study, we evaluated the efficacy and safety of anti-metabolic agent pegaraspargase plus PD-1 antibody sintilimab as chemo-free first-line treatment for advanced stage NKTCL. Besides, exploratory analysis was performed to identify potential biomarkers correlated with treatment response and shed light on future new therapeutic targets in NKTCL.

## Results

### Baseline characteristics

**Fig. 1:**
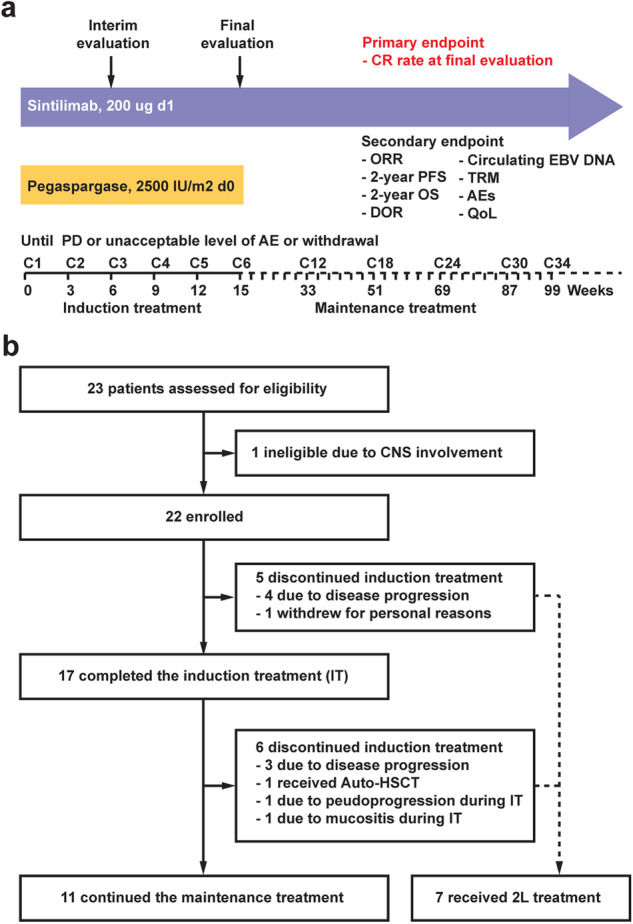
Trial profile. **a** Study schema. Patients received pegaspargase 2500 IU/m^2^ intramuscularly on day 1 and sintilimab 200 mg intravenously on day 2 for 6 cycles of induction treatment, as well as sintilimab 200 mg for 28 cycles of maintenance treatment. Treatment was continued until disease progression, unacceptable levels of adverse events, or withdrawal of consent occurred. **b** Flow diagram. Among 22 patients enrolled, 17 completed the induction treatment and 11 continued the maintenance treatment

From June 2020 to August 2022, 23 patients were assessed for eligibility and 22 patients were enrolled (detailed clinical information was listed in Supplementary Table 1). One patient was excluded due to central nervous system involvement (Fig. 1b). Four patients discontinued the induction treatment owing to disease progression. One patient achieved partial response (PR) at interim evaluation but withdrew consent at 5 months for personal reasons, who was confirmed as responder in the intention-to-treat (ITT) analysis. Seventeen patients completed the induction treatment and 13 patients achieved CR, 1 achieved PR and 3 documented as progressive disease (PD) at final evaluation. Among the 14 CR/PR patients, 11 continued maintenance treatment of sintilimab and 8 of them completed the maintenance treatment, one patient received autologous hematopoietic stem cell transplantation (auto-HSCT), two patients did not proceed to maintenance treatment due to pseudoprogression and mucositis during induction treatment, respectively.

Baseline characteristics are listed in Table 1. The median age was 51 years (range, 24–74) with 23% of patients more than 60 years old. 68% had Ann Arbor stage IV disease, 59% had elevated serum lactate dehydrogenase (LDH) levels, and 45% had remote lymph node involvement. Risk stratification showed that 82 and 55% of the patients were categorized into a high-risk group according to the prognostic index for NKTCL (PINK) and PINK in combination with circulating EBV DNA (PINK-E), respectively.

**Table 1:** Characteristics of the patients

Characteristics	Total, *N* (%)	Responder, *N* (%)	Non-responder, N (%)	*P* Value
Age				
≤60	16 (73%)	10 (67%)	6 (86%)	0.3501
>60	6 (27%)	5 (33%)	1 (14%)	
Sex				
Male	16 (73%)	12 (80%)	4 (57%)	0.2622
Female	6 (27%)	3 (20%)	3 (43%)	
B symptoms				
Absence	15 (68%)	11 (73%)	4 (57%)	0.4476
Presence	7 (32%)	4 (27%)	3 (43%)	
Performance status				
ECOG 0/1	11 (50%)	6 (40%)	5 (71%)	0.1697
ECOG 2	11 (50%)	9 (60%)	2 (29%)	
Ann Arbor stage				
III	7 (32%)	4 (27%)	3 (43%)	0.4476
IV	15 (68%)	11 (73%)	4 (57%)	
Involvement of the nasal area				
No	8 (36%)	5 (33%)	3 (43%)	0.6654
Yes	14 (64%)	10 (67%)	4 (57%)	
Involvement of lymph node				
None	6 (27%)	5 (33%)	1 (14%)	0.2474
Regional	6 (27%)	5 (33%)	1 (14%)	
Distant	10 (45%)	5 (33%)	5 (71%)	
Extranodal site				
Nasopharynx	14 (63%)	10 (67%)	4 (57%)	0.5088
Skin	6 (27%)	6 (40%)	0	
Gastrointestinal tract	6 (27%)	4 (27%)	2 (29%)	
Other				
Lung	2 (9%)	1 (7%)	1 (14%)	
Adrenal gland	2 (9%)	1 (7%)	1 (14%)	
Tonsil	2 (9%)	1 (7%)	1 (14%)	
Pericardium/Ventricle	1 (4%)	1 (7%)	0	
Bone	2 (9%)	2 (13%)	0	
Muscle	2 (9%)	2 (13%)	0	
Pleura/peritoneum	2 (9%)	1 (7%)	1 (14%)	
Testis	1 (4%)	1 (7%)	0	
Thyroid gland	1 (4%)	0	1 (14%)	
Pericardium/Ventricle	1 (4%)	1 (7%)	0	
Pelvic cavity	1 (4%)	0	1 (14%)	
Involvement of bone marrow				
No	19 (86%)	13 (87%)	6 (86%)	0.9517
Yes	3 (14%)	2 (13%)	1 (14%)	
Serum LDH				
Normal	9 (41%)	7 (47%)	2 (29%)	0.4214
Increased	13 (59%)	8 (53%)	5 (71%)	
EBV DNA in whole blood				
Non-detectable	10 (45%)	7 (47%)	3 (43%)	0.8673
Detectable	12 (55%)	8 (53%)	4 (57%)	
PINK				
Low	0	0	0	0.3881
Intermediate	4 (18%)	2 (13%)	2 (29%)	
High	18 (82%)	13 (87%)	5 (71%)	
PINK-E				
Low	1 (4%)	0	1 (14%)	0.6951
Intermediate	9 (41%)	7 (47%)	2 (29%)	
High	12 (55%)	8 (53%)	4 (57%)	
HSCT				
Auto-HSCT	2 (9%)	1 (7%)	2 (29%)	0.1632
Allo-HSCT	0	0	0	
No	20 (91%)	14 (93%)	5 (71%)	
≥2 L Regimens		N.A.		N.A.
Sintilimab + MESA	3 (43%)		3 (43%)	
Sintilimab + P-GEMOX	2 (29%)		2 (29%)	
GEMOX	1 (14%)		1 (14%)	
Selinexor + GEMOX	1 (14%)		1 (14%)	
Sintilimab + Selinexor + P-GEMOX	1 (14%)		1 (14%)	
BV + Selinexor + Mitoxantrone	1 (14%)		1 (14%)	
Clinical trial	1 (14%)		1 (14%)	

*P* values were compared between responders and non-responders by chi-square test

2 L, second-line

*MESA* methotrexate, etoposide, dexamethasone, pegaspargase, *SMILE* dexamethasone, methotrexate, ifosfamide, asparaginase, etoposide, *ESA* etoposide, dexamethasone, pegaspargase, *GLIDE* gemcitabine, pegaspargase, isofosfomide, dexamethasone, epotoside, *P-GEMOX* pegaspargase, gemcitabine, oxaliplatin, *GEMOX* gemcitabine, oxaliplatin

### Efficacy

**Fig. 2:**
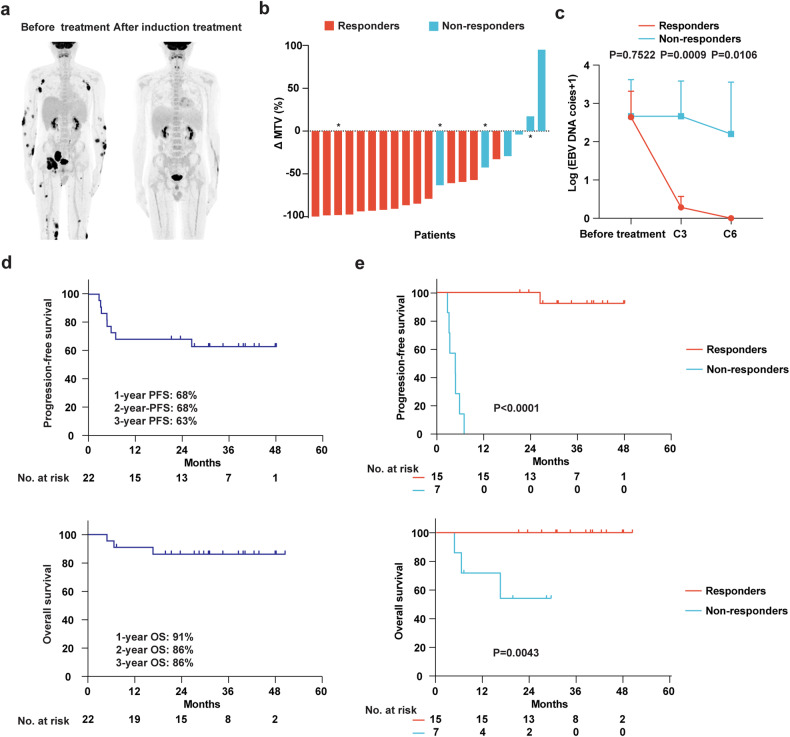
Response and outcomes. **a** Representative PET-CT image of a CR patient before and after induction treatment. **b** Percentage decline of metabolic tumor volume (MTV) during the induction treatment in 21 patients with available PET-CT evaluation. The decline in MTV was measured with interim evaluation of PET-CT in 4 of 22 cases (marked with an asterisk), 3 patients discontinued the treatment, and another withdrew after interim evaluation. The decline in MTV could not be measured in 1 case, who discontinued the treatment before interim evaluation. **c** Dynamical documentation of circulating EBV DNA copies during induction treatment in responders and non-responders. **d** Kaplan-Meier plot showing PFS and OS. The numbers of patients who could be evaluated for each endpoint are shown. Tick marks indicate censored data. **e** Kaplan-Meier plot showing PFS and OS of responders and non-responders in the chemo-free cohort. *P* values in (**c**) were compared between responders and non-responders using student’s *t* test. *P* values in (**e**) were compared between responders and non-responders using log-rank test

In the first stage with 7 patients enrolled, 5 achieved CR and 2 achieved PR. In the ITT analysis, the overall response rate (ORR) was 68% (95%CI, 47–84%) after 6 cycles of induction treatment, with the CR and PR rate as 59 and 9%, respectively. Representative PET-CT images from a CR patient are shown in Fig. 2a. Tumor burden decreased by ≥ 50% in most patients (71%, Fig. 2b). One patient, with the appearance of new lesions in the mediastinum, was diagnosed as pseudoprogression by biopsies (supplementary Fig. 1). Circulating EBV DNA levels were significantly decreased after 3 cycles of induction treatment in responders, as compared to those of non-responders, and turned negative after 6 cycles of induction treatment (Fig. 2c). With a median follow-up of 30 (range, 3–48) months for PFS and 30 (range, 6–50) months for OS, the 2 year PFS and OS rates for the chemo-free cohort were 68% (95%CI, 45–83%) and 86% (95%CI, 63–95%), respectively (Fig. 2d). The median duration of remission (DOR) was not reached. Of note, responders showed better PFS and OS than those of non-responders (Fig. 2e). Considering that upfront auto-HSCT showed no survival benefit,^21,22^ consolidation with auto-HSCT was not generally performed in this trial and only one responder received auto-HSCT. The 13 CR patients remained alive and disease-free under maintenance treatment (*n* = 10) or follow-up evaluation (*n* = 3). Of note, none of them experience recurrence after discontinuation of treatment (median 5 months, range, 0–34 months). One PR patient experienced disease progression after 20 cycles of sintilimab maintenance and remained alive after second-line treatment. The 7 non-responders received ≥2 L chemotherapies (Table 1). with 2 of them achieving CR. One treated with selinexor, sintilimab and P-GEMOX, another treated with sintilimab and MESA.

### Safety

Among 22 eligible patients treated with protocol therapy, 91% (*n* = 20) experienced at least one AE, most of which were mild and moderate. Grade 3/4 AEs were observed in 10 (45%) patients, including neutropenia (32%, *n* = 7), hypofibrinogenemia (18%, *n* = 4), leukopenia (9%, *n* = 2), anemia (5%, *n* = 1), thrombocytopenia (5%, *n* = 1), hypoalbuminemia (5%, *n* = 1), aspartate aminotransferase (AST)/alanine aminotransferase (ALT) elevation (5%, *n* = 1), and heart failure (5%, *n* = 1), which were manageable and led to no discontinuation of treatment. No hypersensitive reaction to pegaspargase occurred and no premedication of corticosteroids was administrated. No bleeding or thromboembolic events occurred. One patient received implantable cardioverter defibrillator surgery due to paroxysmal ventricular tachycardia 6 months before being diagnosed as NKTCL and experienced heart failure after the first cycle of induction treatment. The patient continued the treatment and achieved CR after induction treatment. Therefore, this is considered treatment unrelated.

The most frequent grade 1/2 hematological AEs were anemia (64%, *n* = 14), leukopenia (55%, *n* = 12) and neutropenia (32%, *n* = 7), while non-hematological AEs were hyperbilirubinemia (73%, *n* = 16), hypoalbuminemia (68%, *n* = 15), hypofibrinogenemia (55%, *n* = 12), AST elevation (50%, *n* = 11), and hyponatremia (50%, n = 11).

Eight patients had documented grade 1/2 hypothyroidism (36%), which were asymptomatic and need no medications. No grade 3/4 hypothyroidism was observed. Other immune-related AEs, such as pneumonitis, adrenal insufficiency, hepatitis, vitiligo, etc.,^23^ were not observed. All grade AEs that occurred during induction treatment are listed in Table 2.

**Table 2:** Treatment-related AEs

	Grade 1/2	Grade 3/4
Leukopenia	12 (55%)	2 (9%)
Neutropenia	7 (32%)	7 (32%)
Anemia	14 (64%)	1 (5%)
Thrombocytopenia	2 (9%)	1 (5%)
Hypofibrinogenemia	12 (55%)	4 (18%)
Prolonged activated partial thromboplastin time	10 (45%)	0
Hypoalbuminemia	15 (68%)	1 (5%)
Hyperbilirubinemia	16 (73%)	0
ALT elevation	9 (41%)	1 (5%)
AST elevation	11 (50%)	1 (5%)
Creatine	3 (14%)	0
Hyponatremia	11 (50%)	0
Hyperkalemia	7 (32%)	0
Serum amylase increased	3 (14%)	0
Hyperglycemia	3 (14%)	0
Hypertriglyceridemia	4 (18%)	1 (5%)
Hypothyroidism	8 (36%)	0
Nausea	5 (23%)	0
Vomiting	1 (5%)	0
Diarrhea	0	0
Mucositis	5 (23%)	0
Infection	0	0
Heart failure	0	1 (5%)

### Comprehensive characterization according to treatment response

**Fig. 3:**
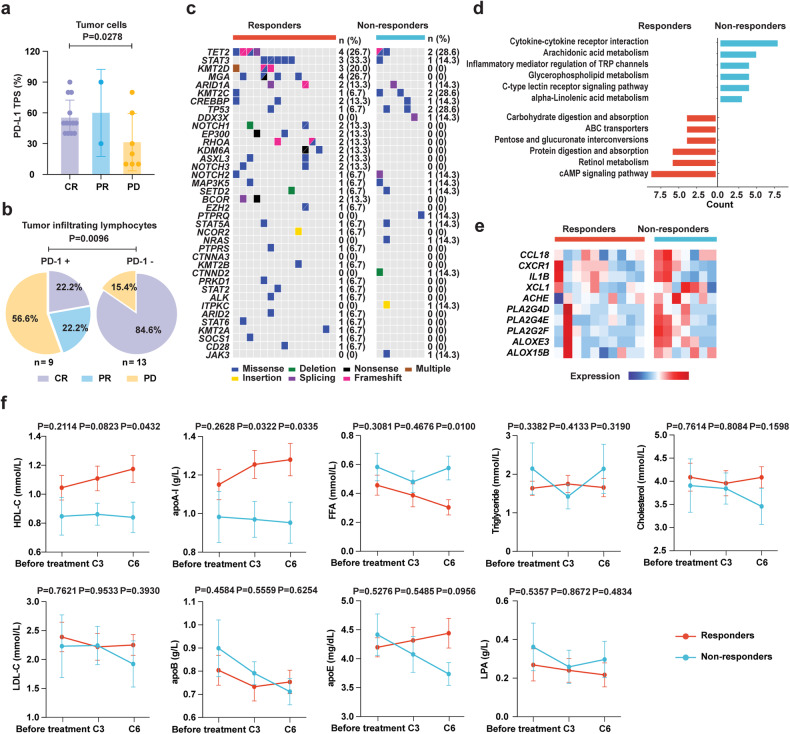
Molecular signatures according to treatment response. **a** PD-L1 expression by tumor proportion score (TPS) according to treatment response. **b** PD-1 expression on tumor infiltrating lymphocytes according to treatment response. **c** Gene mutations identified in responders and non-responders. **d** Pathway enriched with differentially expressed genes in responders and non-responders. **e** Heatmap of genes involved in cytokine interactome and lipid metabolism highly expressed in non-responders. **f** Dynamical documentation of indicated lipoproteins in circulation upon induction treatment in responders and non-responders. Data in (**a**) and (**f**) were represented as mean ± SEM. *P* value in (**a**) was compared between CR and PD patients using student’s *t* test. *P* value in (**b**) was compared between patients with PD-1+ and PD-1- TILs using chi-square test. *P* values in (**f**) were compared between responders and non-responders using student’s *t* test

None of the clinical characteristics was associated with the response to pegaspargase plus sintilimab treatment, such as Ann Arbor stage, extra nasal type, remote lymph node involvement, circulating EBV DNA, PINK, and PINK-E, etc. (Table 1, supplementary Table 2 and Fig. 2a, b). Tumor EBV gene expression before treatment showed no difference between responders and non-responders (supplementary Fig. 2c). PD-L1 expression is detected by PD-L1 immunohistochemical assay and quantified by tumor proportion score (TPS), which is defined by the percentage of stained tumor cells over total tumor cells.^24^ PD-L1 TPS of pre-treatment tumor tissues in CR patients was significantly higher than that in PD patients (Fig. 3a), while PD-1 expression on TIL correlated with poor response to treatment (Fig. 3b).

For exploratory biomarker analysis, we performed targeted DNA sequencing (*n* = 22) and RNA sequencing (RNA-seq, *n* = 17) on pre-treatment tumor samples of the patients. No significant difference in the mutation pattern was shown between responders and non-responders (Fig. 3c and supplementary Table 3). Mutation pattern in newly diagnosed and paired recurrent tumor biopsies was shown in supplementary Fig. 3a. We next compared differentially expressed genes using RNA-seq data and identified 303 upregulated and 519 downregulated genes in non-responders (supplementary Table 4), among which cytokine-cytokine receptor interaction, arachidonic acid, alpha-linolenic acid and glycerophospholipid metabolism were significantly activated in non-responders (Fig. 3d). Similar results were observed by comparing the RNA-seq data of newly diagnosed and paired recurrent tumor biopsies derived from one of the non-responders (supplementary Fig. 3b). Further revealed in Fig. 3e, genes upregulated in non-responders included *CCL8*, *CXCR1*, *IL1B*, and *XCL1* involving recruitment, migration, expansion and function of immunosuppressive Treg cells,^25–28^ as well as *ACHE*, *PLA2G4D*, *PLA2G4E*, *PLA2G2F*, *ALOXE3* and *ALOX15B*, contributing to phosphatidylcholine catabolism and high-density lipoprotein (HDL) homeostasis.^29^ Accordingly, peripheral blood HDL cholesterol (HDL-C) and apolipoprotein A-I (apoA-I), the main protein component of HDL, were elevated, whereas free fatty acids were decreased upon induction treatment in responders (Fig. 3f). Other blood lipids indicators, including triglyceride, cholesterol, low-density lipoprotein cholesterol (LDL-C), apoB, apoE, and lipoprotein A (LPA), showed no difference between responders and non-responders (Fig. 3f). It was worth noting that none of the patients took oral lipid-lowering drugs before treatment and all patients were on a low-fat diet during induction treatment due to administration of pegaspargase.

**Fig. 4:**
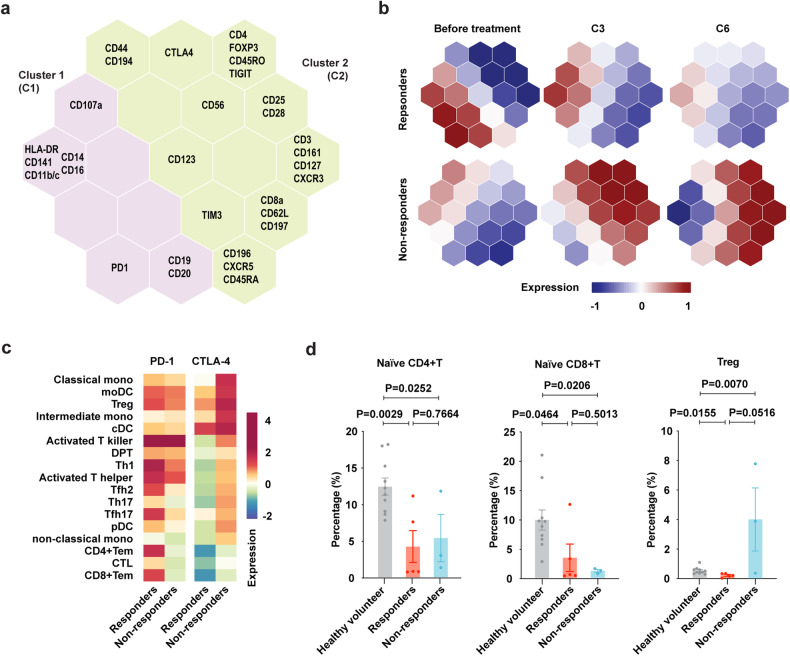
Peripheral immune signatures according to treatment response. SOM plot showing the expression pattern of different cell markers (**a**) upon combination treatment in responders and non-responders (**b**). **c** Heatmap plot of PD-1 and CTLA-4 expression across indicated cell subsets in responders and non-responders. **d** Percentage of distinct cell subsets across healthy volunteers (*n* = 10), responders (*n* = 5) and non-responders (*n* = 3). Data in (**d**) were represented as mean ± SEM. *P* values in (**d**) were compared using student’s *t* test

Inspired by recent studies characterizing the peripheral blood immune signature with response to checkpoint blockade in Hodgkin lymphoma,^20^ we further applied single-cell proteomic analysis (mass cytometry by time-of-flight, CyTOF) to investigate peripheral blood immune signature (*n* = 8, with qualified blood samples) using 40 immune cell markers designating 34 immune cell clusters (supplementary Table 5). As revealed by self-organizing map (SOM)-based gene clustering and visualization, 2 protein clusters (C1 and C2, Fig. 4a) were identified, exerting different expression levels in responders (*n* = 5) and non-responders (*n* = 3). Proteins in C1 (such as HLA-DR, CD141, CD11b, CD11c, CD14, CD16, and CD107a), mainly expressed on monocytes, DCs and B cells, were upregulated in responders, while proteins in C2 (such as FOXP3, CD25, CD127, CD62L, and CD197), mainly expressed on Treg and Naïve T cells, were upregulated in non-responders (Fig. 4b and Supplementary Fig. 4a). Next, we analyzed the protein expression of immune checkpoints and found that PD-1 was increased on T cell clusters in responders, while CTLA-4 was increased on monocytes, DC, and Treg cells in non-responders (Fig. 4c). The proportions of peripheral blood immune cell clusters in NKTCL significantly differed from those of healthy volunteers (*n* = 10, supplementary Fig. 4b, c). Sharing similar features of downregulated naïve CD4^+^ and CD8^+^ T cells in responders and non-responders, Treg cells were downregulated in responders, but upregulated in non-responders (Fig. 4d). Furthermore, peripheral immune cell subsets of all 22 enrolled patients before treatment were assessed by flow cytometry (FCM), confirming a significantly increased CD4^+^CD25^+^CD127^low^ Treg cell subset in non-responders than that of responders (Supplementary Fig. 4d). Immune-suppressive Treg cells were positively correlated with triglyceride, apoE and FFA, while immune-activated cytotoxic T cells were positively correlated with lipid proteins, such as HDL-C, apoA-I, apoB, cholesterol, LDL-C and LPA (Supplementary Fig. 4e).

## Discussion

Currently, there is no standard of care for advanced stage NKTCL, and regimens mainly including SMILE, DDGP, and P-GEMOX are preferred regimens according to the NCCN guidelines.^1^ In two phase 2 studies comprising 125 patients with newly diagnosed advanced stage or relapsed/refractory NKTCL, SMILE regimen is shown to achieve excellent efficacy, with the CR rate and ORR as 53 and 78%, respectively, after 2 cycles of treatment.^9,30^ In another randomized clinical trial comparing the efficacy and safety of SMILE and DDGP for advanced stage and treatment naïve NKTCL, the 3 year PFS rates (56.6% vs 41.8%) and 5 year OS rates (74.3% vs 51.7%) in DDGP were significantly higher than that in SMILE, grade 3/4 hematologic AEs including leukopenia (62.5% vs 85%) and neutropenia (65% vs 85%) were reduced in DDGP than in SMILE.^31^ Two retrospective studies comprising 44 patients with newly diagnosed advanced stage or relapsed/refractory NKTCL treated with P-GEMOX in combination with PD-1 antibody (*n* = 9) or with additional radiotherapy (*n* = 11) or auto-HSCT (*n* = 7) reported CR rate and ORR as 54.5% and 81.8%.^32,33^ To our knowledge, pegaspargase plus sintilimab is the first reported chemo-free regimen in treating newly diagnosed patients with advanced NKTCL. The CR rate and ORR are 59 and 68%, respectively. The therapeutic response was durable with the longest duration as 48 months and the median DOR was not reached. As a “metabolic checkpoint” in NKTCL, glutamine shapes metabolic plasticity of cancer cells and cytotoxic T cells with therapeutic potential.^3^ Co-targeting glutamine metabolism and immune checkpoint, pegaspargase plus sintilimab significantly prolonged survival (2 year PFS as 68% and OS as 86%) with manageable AEs (grade 3/4 leukopenia as 9% and neutropenia as 32%), encouraging further validation in large-scale, multi-center, randomized clinical trial.

The interaction between tumoral PD-L1 and PD-1 on TILs contributes to immune evasion in multiple cancers and is the key therapeutic target of anti-PD1 antibody.^18^ The likelihood of response was not associated with clinical parameters and oncogenic mutations but was associated with the expression of PD-L1 and PD-1. Tumoral PD-L1 expression linked to favorable response to pegaspargase plus sintilimab treatment. As for TILs, tumor-specific T cells (effector) and virus-specific T cells (bystander) informed different immunotherapy responses.^34^ Virus-specific T cells, with the phenotypic traits of PD-1 expression, prevented efficient tumor killing by effector T cells upon PD-1 antibody treatment.^34^ Accordingly, PD-1 expression on TILs was associated with resistance to pegaspargase plus sintilimab in NKTCL.

CyTOF analysis defined peripheral immune signatures, revealing significantly reduced naïve CD4^+^ and CD8^+^ T cells in NKTCL patients than that in healthy volunteers. Similar results were reported in classical Hodgkin lymphoma^20^ and non-small cell lung cancer (NSCLC).^35^ As for treatment responses, a significantly increased proportion of Treg cells before treatment were identified in non-responders, as evidenced by CyTOF and FCM analysis. Besides, CTLA-4, which inhibits T-cell activation through potentially synergistic mechanisms with PD-1, was highly expressed in peripheral Treg cells of non-responders. Anti-CTLA-4 antibody exerts anti-tumor effects through blocking the CTLA-4-mediated immunosuppressive function of Tregs and depleting Tregs by Fc-mediated antibody-dependent cellular cytotoxicity and phagocytosis.^36^ Indeed, dual targeting PD-1 and CTLA-4 showed promising efficacy and favorable tolerability in advanced stage solid tumors like cervical cancer and NSCLC.^37,38^

Lipids promote tumorigenesis, colonization, and metastasis of tumor cells, and modulate the pro-tumor activity of immune cells, like Treg cells, M2 macrophages, and myeloid-derived suppressor cells.^39^ Lipoproteins (such as HDL-C, LDL-C, and LPA) are primary mediators of lipid metabolites (such as fatty acid, triglyceride, and cholesterol) transport.^40^ Apolipoproteins (such as apoA-I, apoB, and apoE) are essential components of lipoproteins and ligands for lipoprotein receptors.^40^ HDL and apoA-I, harboring antitumorigenic activities through decelerating oxidative stress and inflammation in tumor microenvironment,^41^ were significantly upregulated in responders upon treatment. FFA, suppressing cancer immunogenicity by activating Treg cell maturation,^42,43^ was upregulated in non-responders upon treatment. Enhanced fatty acid metabolism characterized the metabolic phenotype of multiple cancers and could potentially be targeted for therapeutic intervention.^44^ Recent studies have reported that inhibition of fatty acid metabolism sensitized tumor cells to various existing anti-tumor agents.^45,46^

Metabolic reprogramming, immune suppression, epigenetic dysregulation, cell-cycle progression, cell death resistance and sustaining proliferation were hallmark characteristics during the pathogenesis of NKTCL.^47^ Pegasparagase-containing chemotherapies, dual targeting metabolic reprogramming and cell-cycle progression, showed efficacy in treating patients relapsed from or refractory to chemo-free pegarspargase and sintilimab regimen. In addition to malignant transformation, epigenetic dysregulation contributed to exhausted T cell phenotype,^48^ indicating therapeutic potential of checkpoint blockade in combination with epigenetic modifiers. With the expanding knowledge on the pathogenic mechanism of NKTCL, a plethora of novel treatment strategies, including cell-surface-targeted antibodies, chimeric antigen receptor T cells, EBV-specific T lymphocyte, signaling pathway inhibitors and epigenetic drugs,^47^ are under development for future molecular signature-guided targeted therapies.

Due to the rarity of advanced stage NKTCL, one limitation of the study is the modest sample size and relatively short follow-up. Nevertheless, the optimal response rate and survival observed in this study have provided substantial evidence to initiate a multicenter, prospective, randomized clinical trial (NCT06255795) to compare the efficacy and safety of pegaspargase and sintilimab with current first-line chemotherapy for newly diagnosed advanced stage NKTCL.

In conclusion, first-line chemo-free pegaspargase and sintilimab regimen, co-targeting glutamine metabolism and immune checkpoint PD-1, is effective and safe in treating newly diagnosed, advanced stage NKTCL. Modulating tumor metabolism and immunosuppressive status may pave the future pathway of mechanism-guided treatment in NKTCL.

## Material and methods

### Study design and participants

The pegaspargase plus sintilimab regimen in advanced stage and treatment naïve NKTCL was an investigator-initiated, open-label, single-arm, phase 2 study. All patients were histologically diagnosed according to the World Health Organization (WHO) classification and reviewed by two independent pathologists (C.-F.W. and H.-M.Y.). Eligible patients had an age of 18 years or older, an Eastern Cooperative Oncology Group (ECOG) performance-status score of 2 or less, and adequate renal and hepatic function and function. This study was approved by the ethics committee and institutional review board of Shanghai Ruijin Hospital. In accordance with the Declaration of Helsinki and Good Clinical Practice guidelines, informed consent was obtained from all patients.

### Treatment and assessments

As revealed in Fig. 1a, enrolled patients were treated with pegaspargase 2500 IU/m^2^ intramuscularly on day 1 and sintilimab 200 mg intravenously on day 2 for 6 cycles of 21 days (C1-C6, induction treatment), as well as sintilimab 200 mg for 28 cycles of 21 days (C7-C34, maintenance treatment). No premedication, such as corticosteroids, was administrated. Radiation was not allowed during induction treatment. During induction treatment, prophylactic intrathecal methotrexate 10 mg and cytarabine 50 mg for 4 cycles were administrated in patients with involvement of bone marrow, nasal or paranasal sinuses, orbit, breast, kidney, adrenal gland, or testis. At the end of induction treatment, radiation was administrated in patients with limited lesions or residual disease according to Investigators’ consideration. Treatment continued until disease progression, unacceptable AE, withdrawal of consent, investigator decision, or until completion of the planned treatment regimen.

All patients underwent pre-treatment evaluation with positron emission tomography-computed tomographic (PET-CT) and bone marrow aspiration and biopsy. Enhanced magnetic resonance imaging (MRI) of the head and neck was performed for patients with involvement of the nasopharynx. PET-CT and enhanced MRI were repeated after C3 (interim evaluation), C6 (final evaluation), C18 and C34. Enhanced CT of the neck, thorax, abdomen, and pelvis was performed after C12, C24, and C30. Response assessments were performed according to the Lugano classification.^49,50^ Treatment continued If documented as CR, PR, or stable disease (SD). “Indeterminate response (IR)” was introduced to identify lesions with imaging findings of PD despite evidence of clinical benefit, until confirmed as pseudoprogression or true PD according to biopsy or subsequent imaging.^49^ Bone marrow aspiration was repeated at interim and final evaluation for patients with evidence of bone marrow involvement.

Toxic effects were graded based on the Common Terminology Criteria for Adverse Events, version 4.03. Blood counts and biochemical tests, including hepatic, renal, and thyroid function, coagulation indicators, glucose, lipase, and EBV DNA in whole blood, etc. were conducted every 1–2l weeks during treatment and every 3 months thereafter. In cases of grade 3 hematologic and immune toxicities, treatment was withheld until resolving to grade 0 or 1. If grade ≥ 3 neutropenia was present in the first cycle of chemotherapy, G-CSF prophylaxis with pegfilgrastim 6 mg subcutaneously was administrated from the second cycle. If grade ≥ 3 hypofibrinogenemia was present, fibrinogen was administrated.

### Endpoints

The primary endpoint was the CR rate at the final evaluation of induction treatment. The secondary endpoints included ORR, which was defined as the percentage of CR/PR patients, 2 year PFS, which was calculated from the date of enrollment to the date of disease progression or death from any cause, 2 year OS, which was calculated from the date of enrollment to final follow-up or death from any cause, DOR, circulating EBV DNA, treatment-related mortality (TRM), AEs and quality of life (QoL). To identify the biomarkers associated with treatment response, we performed in patients with qualified samples, targeted DNA sequencing and RNA-seq on pre-treatment tumor samples, as well as CyTOF and FCM analysis on blood samples.

### Statistical analyses

Among previously reported chemotherapeutic regimens for advanced stage and treatment naïve NKTCL (mainly including SMILE, DDGP, and P-GEMOX), the best CR rate was 71% (15/21) for DDGP.^10^ Considering that pegaspargase plus sintilimab was a chemo-free regimen, we therefore set the target CR rate as 70%, which is non-inferiority to the current best CR rate. The sample size was calculated to reject the null hypothesis of 40% CR rate^9^ and favor a target CR rate of 70%, with a significance of 0.05 and power of 80% using Simon’s Minimax 2-stage design.^51^ Thus, this study continued if the CR rate was >3/7 during the first stage. If the CR rate is >3/7 during the first stage, the CR rate is >11/20 during the second stage, we reject the null hypothesis and claim that the treatment is promising. The final target number would be 22 given the 10% dropout rate. The chi-square test was used to analyze the association of molecular and laboratory parameters with treatment response. The Wilcoxon rank-sum 2-tailed test was used for comparison of gene expression profiles in responders and non-responders. Survival was analyzed based on Kaplan-Meier curves and compared using the log-rank test. Two-sided statistical tests yielding *P* < 0.05 were considered significant. Statistical analyses were performed using the IBM PASW version 24.0 software program (SPSS Inc., Chicago, IL).

### Supplementary information

SI-R2

Table S1

Table S2

Table S3

Table S4

Table S5

Data S1

Data S2

## Data Availability

The data reported in this article have been deposited in the NODE (http://www.biosino.org/node) platform (accession number OEP003404).
