# Supplementary material for: Anti-metabolic agent pegaspargase plus PD-1 antibody sintilimab for first-line treatment in advanced natural killer T cell lymphoma
Source: Signal Transduct Target Ther. 2024 Mar 6;9:62. doi: 10.1038/s41392-024-01782-8 (PMC10917752; doi:10.1038/s41392-024-01782-8)
Supplement: Supplementary file 1 — SI-R2 [file 41392_2024_1782_MOESM1_ESM.docx]

Supplementary Materials for

Pegarspargase plus sintilimab for first-line treatment in advanced natural killer T cell lymphoma

Jie Xiong^1#^, Shu Cheng^1#^, Xiao Gao^1#^, Shan-He Yu^1^, Yu-Ting Dai^1^, Xin-Yun Huang^2^, Hui-Juan Zhong^1^, Chao-Fu Wang^3^, Hong-Mei Yi^3^, Hao Zhang^4^, Wei-Guo Cao^5^, Rong Li^6^, Wei Tang^1^, Yan Zhao^1^, Peng-Peng Xu^1^, Li Wang^1,7^, Wei-Li Zhao^1,7*^

^1^ Shanghai Institute of Hematology, State Key Laboratory of Medical Genomics, National Research Center for Translational Medicine at Shanghai, Ruijin Hospital Affiliated to Shanghai Jiao Tong University School of Medicine, Shanghai, China

^2^ Department of nuclear medicine, Shanghai Ruijin Hospital, Shanghai Jiao Tong University School of Medicine, Shanghai, China

^3^ Department of Pathology, Shanghai Ruijin Hospital, Shanghai Jiao Tong University School of Medicine, Shanghai, China

^4^ Department of Otolaryngology, Shanghai Ruijin Hospital, Shanghai Jiao Tong University School of Medicine, Shanghai, China

^5^ Department of Radiation, Shanghai Ruijin Hospital, Shanghai Jiao Tong University School of Medicine, Shanghai, China

^6^ Department of Hematology, Navy Medical Center of PLA, Shanghai, P. R. China

^7^ Pôle de Recherches Sino-Français en Science du Vivant et Génomique; Laboratory of Molecular Pathology; Shanghai, China

^#^ These authors have contributed equally.

Correspondence to: [zhao.weili@yahoo.com](mailto:zhao.weili@yahoo.com) (Wei-Li Zhao)

**This PDF file includes:**

Figures. S1 to S4

**Other Supplementary Materials for this manuscript include the following:**

Table S1 to S5

Data S1 to S2

Figure. S1.

**
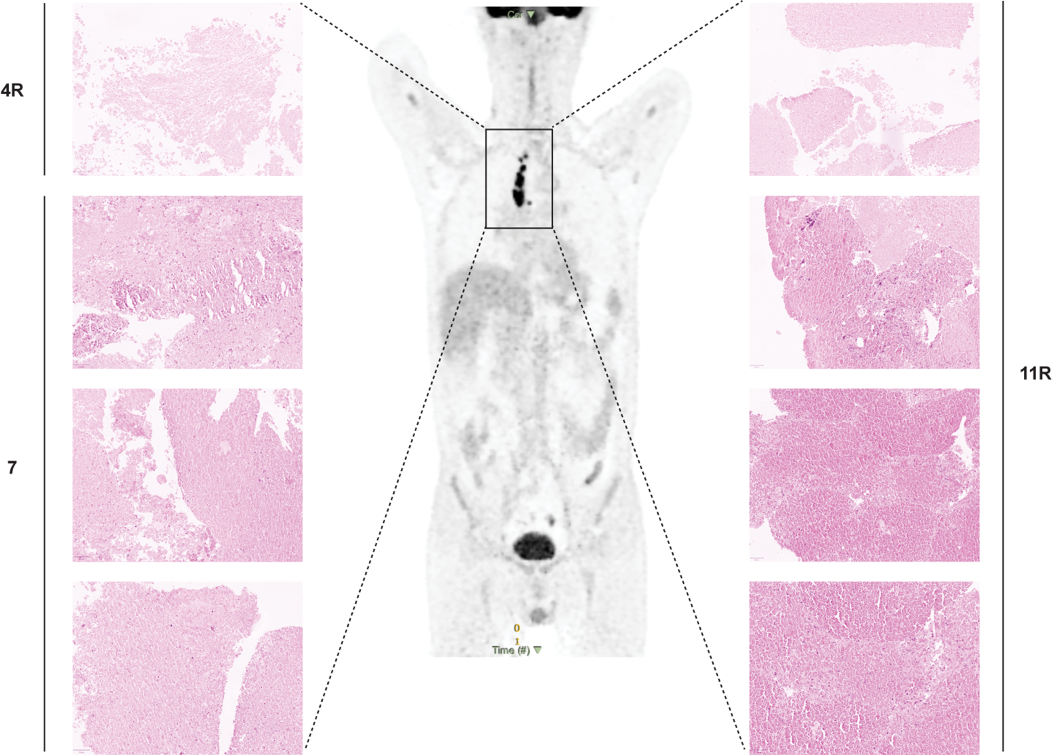
**

**Fig. S1. PET-CT and haematoxylin-eosin (HE) staining image of the patient diagnosed as pseudoprogression at interim evaluation.**

Black box: mediastinal lymph nodes with increased FDG uptake

HE staining of 4R, 7, 11R lymph node biopsies

Figure. S2.


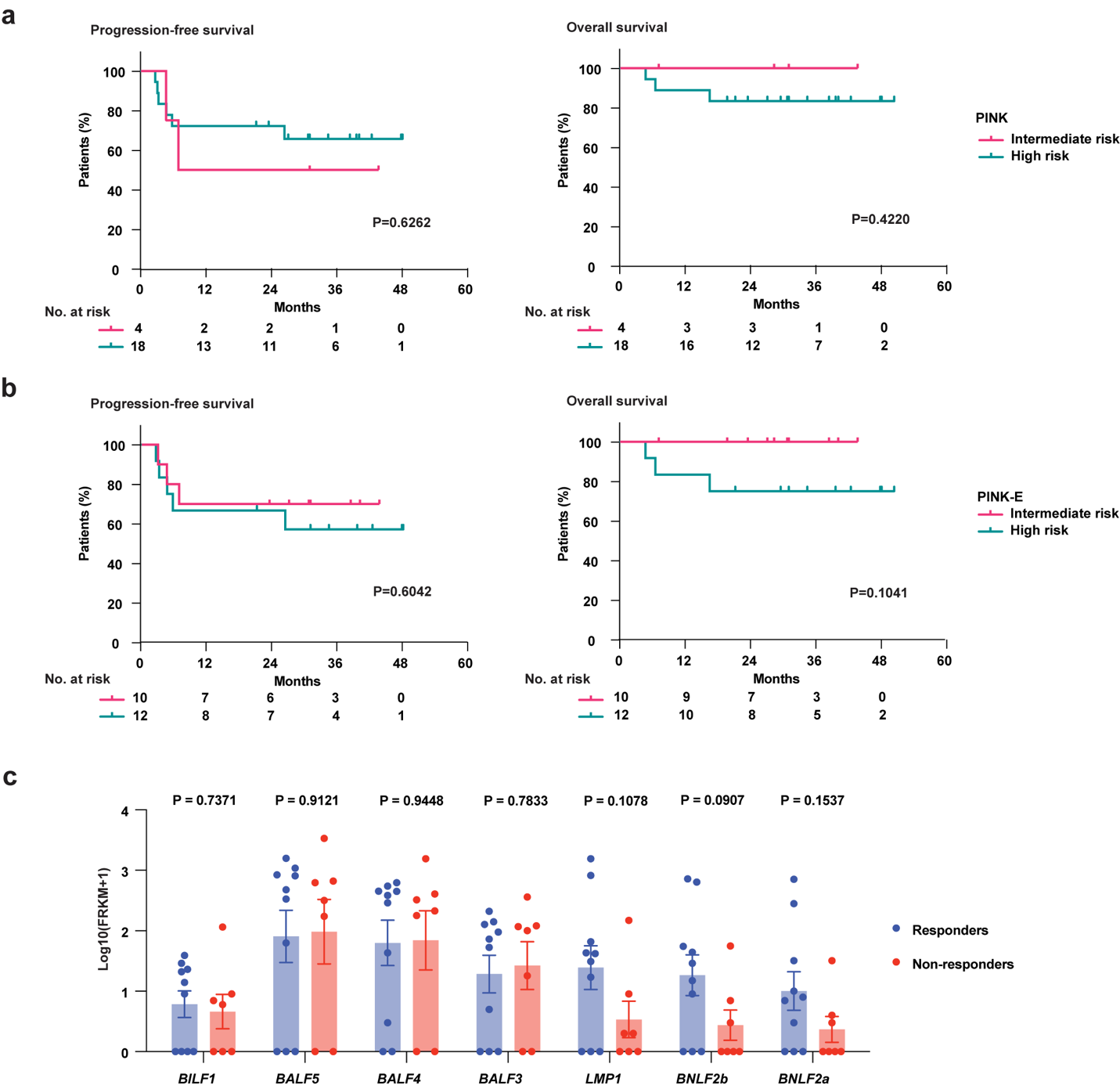


**Fig. S2. Clinical and biological characteristics according to survival or treatment response.**

(a) Kaplan-Meier plot showing PFS and OS according to PINK in chemo-free cohort.

(b) Kaplan-Meier plot showing PFS and OS according to PINK-E in chemo-free cohort.

(c) Indicated EBV gene expression in responders (n = 10) and non-responders (n = 7) assessed by RNA-seq.

P values in (a) and (b) were compared between intermediated risk and high risk group according to PINK and PINK-E, respectively, using log-rank test. P values in (c) were compared between responders and non-responders using student’s t-test.

Figure. S3.

**
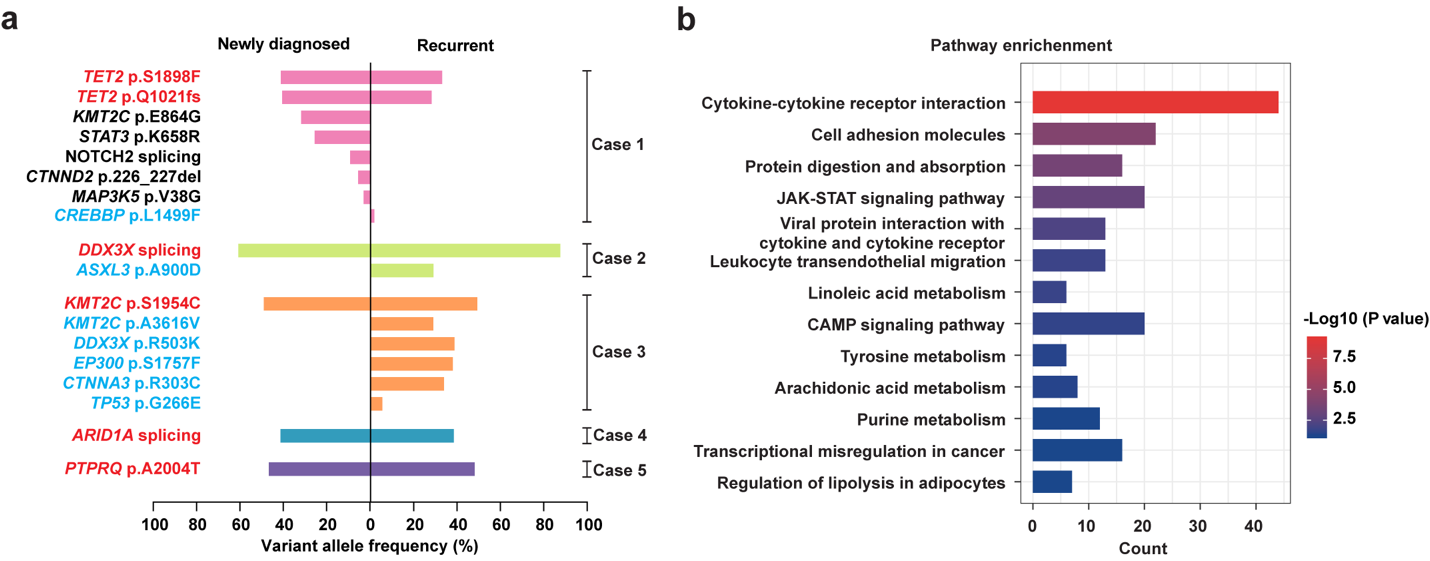
**

**Fig. S3. Molecular signatures of newly diagnosed and paired recurrent NKTCL biopsies.**

(a) Gene mutations identified in newly diagnosed and paired recurrent tumor biopsies.

(b) Pathway enriched with differentially expressed genes in newly diagnosed and paired recurrent tumor biopsies.

Figure. S4.


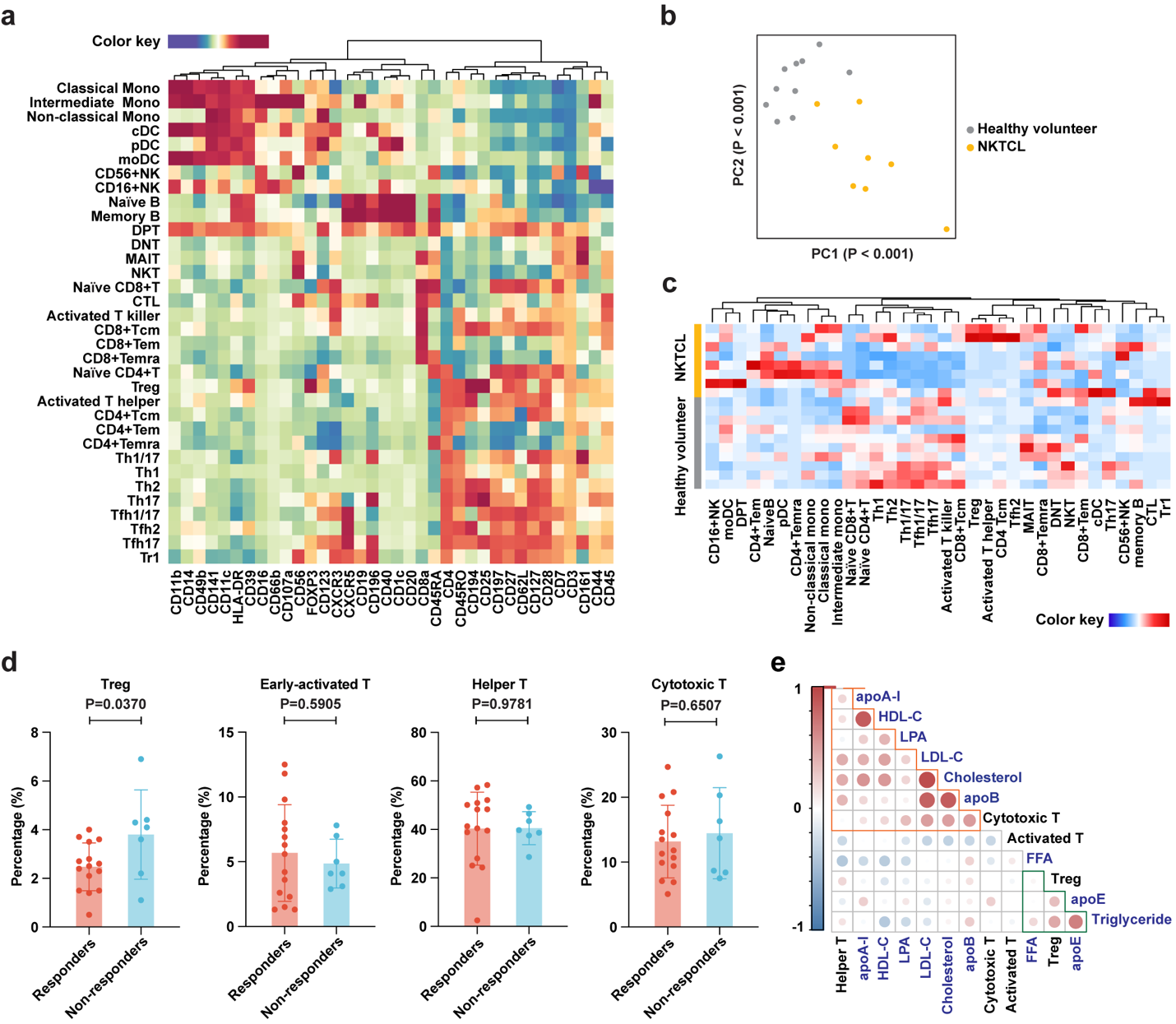


**Fig. S4. Peripheral immune cell composition in healthy volunteer and NKTCL.**

(a) Heatmap plot of normalized expression of markers across distinct immune cell subsets.

(b) PCA plot based on the variation of marker expression across indicated samples.

(c) Heatmap showing percentage of indicated immune cell subsets in healthy volunteer and NKTCL.

(d) Percentage of Treg (CD4+CD25+CD127low), activated T (CD3+CD68+), Helper T (CD4+CD28+), and Cytotoxic T (CD8+CD28+) cell subsets in responders (n = 15) and non-responders (n = 7) assessed by FCM.

(e) Correlation among indicated immune cells and lipid metabolites.

P value in (d) was compared between responders and non-responders using student’s t-test.

Table S1. (separate file)

Clinical information of NKTCL patients (n=22) and healthy volunteers (n=10).

Table S2. (separate file)

Univariant analysis of clinical characteristics according to PFS and OS (n=22).

Table S3. (separate file)

Mutation pattern in responders (n=15) and non-responders (n=7).

Table S4. (separate file)

Differentially expressed genes between respoders (n=10) and non-responders (n=7).

Table S5. (separate file)

List of antibodies used in CyTOF analysis.

Data S1. (separate file)

STROBE checklist

Data S2. (separate file)

Investigator’s brochure
