# Supplementary material for: Anti-metabolic agent pegaspargase plus PD-1 antibody sintilimab for first-line treatment in advanced natural killer T cell lymphoma
Source: Signal Transduct Target Ther. 2024 Mar 6;9:62. doi: 10.1038/s41392-024-01782-8 (PMC10917752; doi:10.1038/s41392-024-01782-8)
Supplement: Supplementary file 8 — Data S2 [file 41392_2024_1782_MOESM8_ESM.pdf]

Clinical Study Protocol

**Protocol Title**	**The Efficacy and Safety of Anti-PD-1 Antibody in Combination With Pegaspargase in the Treatment of Newly Diagnosed, Stage III to IV Extranodal Natural Killer/T-Cell Lymphoma, Nasal Type**
**Date of protocol**	October 15, 2018
**Protocol Code**	NHL-008
**Study Type**	Interventional
**Sponsor**	Shanghai Rui-Jin Hospital,　Shanghai Jiao Tong University School of Medicine<br>197 Ruijin Er Road, Huangpu district<br>Shanghai 200025 (China)
**Principal investigator**	Dr. Weili Zhao, MD<br>197 Ruijin Er Road, Huangpu district<br>Shanghai Rui Jin Hospital,Shanghai Jiao Tong University School of Medicine<br>Shanghai 200025 (China)
**ClinicalTrials.Gov Number**	NCT04096690

1. PROTOCOL SYNOPSIS

SPONSOR	Shanghai Rui-Jin Hospital, Shanghai Jiao Tong University School of Medicine
PROTOCOL TITLE	**The Efficacy and Safety of Anti-PD-1 Antibody in Combination with Pegaspargase in the Treatment of Newly Diagnosed, Stage III to IV Extranodal Natural Killer/T-Cell Lymphoma, Nasal Type**
SHORT TITLE / PROTOCOL ID	Anti-PD-1 Antibody Combined with Pegaspargase in the Treatment of Advanced Stage NK/T-cell Lymphoma /NHL-008
PROTOCOL VERSION	October 15, 2018
TRIAL REGISTRATION	NCT04096690
CLINICAL PHASE	II
INDICATION	Untreated patient with advanced stage NK/T cell Lymphomas
BACKGROUND AND RATIONALE	Nasal natural killer (NK)/T-cell lymphoma is a unique type of non-Hodgkin lymphoma (NHL) that is almost always associated with Epstein-Barr virus (EBV) and shows a geographic predilection for Asian and South American populations. Extranodal NK/T cell lymphoma (NKTCL), nasal type, is a distinct subtype of lymphoma which predominantly occurs in extranodal sites including nasal/paranasal area, and less frequently in skin, gastrointestinal tract and other organs. In patients with disease stage III-IV, the 5-year overall survival (OS) rate is 50%.<br>Aberrant glutamine metabolism is involved in the pathogenesis of NKTCL. Asparaginase exerts an anti-metabolic effect by depleting extracellular asparagine and inhibiting glutamine-dependent tumor cell growth. Asparaginase-containing chemotherapy achieves high efficacy in patients with NKTCL. First-line intensive chemotherapy like SMILE (dexamethasone, methotrexate, ifosfamide, asparaginase, and etoposide) has a complete response (CR) rate of 40% (8/20) in advanced stage NKTCL, which should be safely administrated with careful attention to adverse effects (AEs).<br>NKTCL cells express programmed death protein ligand 1 (PDL1) and the EBV latent membrane protein 1 (LMP1) upregulates PDL1 expression through the MAPK/NF-κB pathway. Ligation of PD1 on effector T-cells with PDL1 on

	lymphoma cells leads to inhibition of T-cell activity, providing a potential mechanism for NKTCL to evade immunosurveillance. Pembrolizumab, a humanized anti-PD-1 monoclonal antibody, exhibited activity in Hodgkin lymphoma (HL). This antibody has revealed to be highly effective for relapsed/refractory NKTCL failing asparaginase-containing therapy. In a recent study seven patients with relapsed NKTCL failing previous L-asparaginase regimens (N = 7) and allogeneic haematopoietic stem cell transplantation (HSCT) (N = 2) were treated with the anti-PD1 antibody pembrolizumab and all seven patients responded, with five complete and two partial responses. Sintilimab, another fully humanized anti-PD-1 antibody with increased affinity to PD-1 than that of pembrolizumab, is implicated in the treatment of NKTCL, HL and solid tumors.<br>Our aim is to conduct a clinical trial to evaluate the activity and tolerability of the anti PD1 agent sintilimab in combination with pegaspargase for the initial treatment of previously untreated patients with advanced stage NKTCL. This is a preparatory study for a larger future randomized study.
OBJECTIVES	<u>Primary Objective</u><br>To test efficacy of anti-PD-1 antibody in combination with pegaspargase in patients with advanced stage NK/T cell lymphoma<br><br><u>Secondary Objectives</u><br>To evaluate the feasibility and safety of anti-PD-1 antibody in combination with pegaspargase in patients with advanced stage NK/T cell lymphoma
ENDPOINTS:	<u>Primary endpoint</u><br>• Complete response rate (CRR defined according to 2014 Lugano criteria and 2016 Refinement of the Lugano Classification lymphoma response criteria in the era of immunomodulatory therapy.)<br>[Time Frame: At the end of Cycle 6 (each cycle is 21 days)]<br><u>Secondary endpoints</u><br>• Overall response rate (ORR defined according to 2014 Lugano criteria and 2016 Refinement of the Lugano Classification lymphoma response criteria in the era of immunomodulatory therapy.)<br>[Time Frame: At the end of Cycle 6 (each cycle is 21 days)]<br>• 2-year progression free survival<br>• 2-year overall survival<br>• Duration of response

	• EBV-DNA load change<br>• Rate of adverse events coded according to CTCAE ver 4.0<br>• QoL<br>• Treatment-related mortality
STUDY DESIGN	This is an investigational, Phase II, Open label, single arm, clinical trial.
INCLUSION/EXCLUSION CRITERIA	<u>Inclusion criteria</u><br>1) Confirmed histological diagnosis of NKTCL nasal type<br>2) No previous anti-lymphoma treatment<br>3) Age > 18 years<br>4) Ann Arbor stage III-IV<br>5) At least one measurable/evaluable site after diagnostic biopsy before treatment start<br>6) ECOG performance status of 0-2<br>7) Adequate hematological and organ function; i.e. ANC >1000 cells /mmc, platelet counts > 50.000/mmc, Hemogolbin > 9 g/dl<br>AST, ALT <3 x ULN; serum bilitubin < 1.5x ULN (patient with Gilbert diasease can be enrolled)<br>Serum creatinine < 2 x ULN or creatinine clearance > 50ml/min<br>8) Tumor tissue (fresh preferred, archivial tissue is also acceptable)<br>9) For women of childeberaing potential a negative pregnancy test on day 1 of cycle 1 and agree to adopta adequate measure to avoid pregnancy during study treatment and for at least one year from EOT.<br>10) For men agreement to remain abstinent or to use barrier contraception<br>11) Signed Informed consent<br><u>Exclusion criteria</u><br>1) Confirmed histological diagnosis of aggressive NK cell leukemia<br>2) Early stage disease (AA stage I-II)<br>3) Evidence of suspect of CNS disease.<br>4) Has an active autoimmune disease that has required systemic treatment in past 2-years (ie, with use of disease modifying agents, corticosteroids or immunosuppressive drugs), including but not limited to myatenia gravis, myositis, autoimmune hepatitis, systemic lupus erythematosus, rheumatoid arthritis, inflammatory bowel disease, vascular thrombosis associate with antiphospholipid syndrome, wegener's granulomatosis, Sjogren syndrome, guillan barreè syndrome, multiple sclerosis, vasculitis or

	glomerulonephritis. The following exception are allowed: patients with autoimmune related hyopthiroidism or type I diabetes mellitus who are on stable treatment. Replacement therapy (eg, thyroxine, insulin, or physiologic corticosteroid replacement therapy for adrenal or pituitary insufficiency) is not considered a form of systemic treatment and is allowed.<br>5) Treatment with systemic immunosuppressive medications, including prednisone, cyclophosphamide, azathioprine, methotrexate, thalidomide and anti tumor necrosis factor (anti-TNF) agents within 2 weeks prior to cycle 1 day 1; inhaled corticosteroids are allowed.<br>6) Active infection requiring systemic therapy<br>7) History of (non-infectious) pneumonitis that required steroids; evidence of interstitial lung disease or active, non-infectious pneumonitis<br>8) Significant cardiovascular disease, myocardial infarciotn in the previous 3 months, unstable arrhythmias, or unstable angina.<br>9) History of other(s) inflitrating cancer(s) in the previous 3 years that were not treated with curative intent or who are still receiving anticancer therapy (including hormone therapy for breast or prostate cancer).<br>10) HBsAg, HCV or HIV positivity. Positive serology is admitted for HBV and HCV but DNA/RNA test must be negative<br>11) Prior therapy with an anti-PD-1, anti-PD-L1, or anti-PD-L2 agent<br>12) Pregnant or lactating women<br>13) Administration of a live attenuated vaccine within 4 weeks before cyle 1 day 1. Patients must not receive live, attenuate vaccines, including influenza vaccines at any time during study.<br>14) Other uncontrollable medical condition that may that may interfere the participation of the study
MEASUREMENTS AND PROCEDURES	All eligible patients will receive induction treatment for six cycles of Anti-PD-1 antibody sintilimab plus pegaspargase (21-day cycle) and sintilimab monotherapy maintenance treatment for about 2 years (21-day cycle).<br>**Pegaspargase**<br>Pegaspargase 2500IU/ m2 administered by intramuscular injection on Day 1 of each 21-day cycle for 6 cycles in induction treatment<br>**Sintilimab**

	Sintilimab 200mg administered intravenously on Day 2 of each 21-day cycle for 6 cycles in induction treatment.<br>**Sintilimab maintenance**<br>Sintilimab 200mg administered intravenously on Day 1 of each 21-day cycle for up to 28 cycles in maintenance treatment.<br><u>Response assessment</u><br>Response will be evaluated according to 2014 Lugano criteria and 2016 Refinement of the Lugano Classification lymphoma response criteria in the era of immunomodulatory therapy using CT scan and PET-CT scan.
NUMBER OF PARTICIPANTS WITH RATIONALE	22 patients
STATISTICAL CONSIDERATIONS	Sample size is defined considering CRR as primary study enpoint. Based on the publication by Yamaguchi et al. CRR for newly diagnosed NKTCL with stage III-IV was 40% for regimen of SMILE. Thus, 40% CRR from published study defines null hypothesis (H0) for our trial. The sample size of 20 pts will allow to demonstrate a 30% improvement of CRR from 40% (null hypothesis, H0) with a type 1 error rate=0.05 (two-sided) and power=0.80. Sample size will be 22 under consideration of drop off rate for 2 subjects.
GCP STATEMENT	This study will be conducted in compliance with the protocol, the current version of the Declaration of Helsinki, the ICH-GCP as well as all national legal and regulatory requirements.

2. TABLE OF CONTENTS

1. PROTOCOL SYNOPSIS 2
2. TABLE OF CONTENTS 7
3. BACKGROUND INFORMATION 9
3.1 NK T cell Lymphomas 9
4. STUDY OBJECTIVES 11
4.1 Primary Objective 11
4.2 Secondary Objectives 11
5 STUDY OUTCOMES 12
5.1 Primary Outcome 12
5.2 Secondary Outcomes 12
6. STUDY DESIGN AND STUDY POPULATION 13
6.1 General Study Design 13
6.2 Eligibility Criteria 14
6.2.1 Inclusion Criteria 14
6.2.2 Exclusion Criteria 14
6.2.3 Criteria for Withdrawal / Discontinuation of Participants 15
7. STUDY INTERVENTION 16
7.1 Induction phase 16
7.2 Maintenance phase 16
7.3 Prophylaxis for CNS relapse and Radiation 16
7.4 Instructions for Initiation of a New Cycle 16
7.5 Management and monitoring of AEs 16
7.6 Concomitant therapy 21
8 STUDY ASSESSMENTS 22
8.1 Schedule of Events 22
8.2 Assessment of Tumor response 22
8.3 Adverse Event Reporting Requirements 23
8.3.1 Assessment of AEs 23
8.3.1 Recording of AEs 23
9. STATISTICAL CONSIDERATIONS 25
9.1 Sample Size Estimation 25
9.2 Analysis Plan 25
9.2.1 Efficacy Analysis 25
9.2.2 Safety Analysis 25

10. REFERENCES 26
APPENDIX 1 - REVISED CRITERIA FOR RESPONSE ASSESSMENT (2014) 28
APPENDIX 2 - RESOPONSE CRITERIA IN THE ERA OF IMMUNOMODULATORY THERAPY (2016) 30
APPENDIX 3 - ECOG PERFORMANCE STATUS SCALE 31
APPENDIX 4: ANN ARBOR STAGING 32
APPENDIX 5 - RECOMMENDED TREATMENT MODIFICATIONS FOR SINTILIMAB 33

3. BACKGROUND INFORMATION

3.1 NK T cell Lymphomas

Nasal natural killer (NK)/T-cell lymphoma is a unique type of non-Hodgkin lymphoma (NHL) that is almost always associated with Epstein-Barr virus (EBV) and shows a geographic predilection for Asian and South American populations[1]. Extranodal NK/T cell lymphoma (NKTCL), nasal type, is a distinct subtype of lymphoma which predominantly occurs in extranodal sites including nasal/paranasal area, and less frequently in skin, gastrointestinal tract and other organs [2]. In patients with disease stage III-IV, the 5-year overall survival (OS) rate is 50% [3].

Aberrant glutamine metabolism is involved in the pathogenesis of NKTCL. Asparaginase exerts an anti-metabolic effect by depleting extracellular asparagine and inhibiting glutamine-dependent tumor cell growth[4]. Asparaginase-containing chemotherapy achieves high efficacy in patients with NKTCL[5]. First-line intensive chemotherapy like SMILE (dexamethasone, methotrexate, ifosfamide, asparaginase, and etoposide) has a complete response (CR) rate of 40% (8/20) in advanced stage NKTCL, which should be safely administrated with careful attention to adverse effects (AEs) [6].

Tumor immune escape is an emerging hallmark of cancer and an important target for cancer therapy. Programmed cell death receptor 1 (PD-1) and PD ligand 1 (PD-L1) are important immune checkpoint molecules involved in T cell-mediated immune response and are key regulators of tumor immune escape[7, 8]. PD-L1, also known as B7-H1, is an immunomodulatory cell-surface glycoprotein and a member of the B7 family of costimulatory molecules that is primarily expressed by antigen-presenting cells and serves to regulate the cellular immune response. Binding of PD-L1 to its cognate receptor PD-1 inhibits proliferation of activated T cells in peripheral tissues leading to “T-cell exhaustion”, a functional phenotype that can be reversed by PD-1 blockade[9]. Aberrant expression of PD-1/PD-L1 on tumor cells or tumor-infiltrating lymphocytes has conferred adverse prognostic impact in multiple solid and hematopoietic malignancies[10]. Blockade of the PD-1/PD-L1 interactions with monoclonal antibodies has achieved encouraging efficacy and has been approved by the US Food and Drug Administration (FDA) in many malignancies including Hodgkin lymphoma (HL) and primary mediastinal large B-cell lymphoma (PMBL)[20]. In HL and PMBL, the gene amplification of the 9p24.1 locus (PD-1 ligands and JAK2) promotes PD-L1 expression via the JAK2-STAT signaling pathway in a dose-dependent manner[11]. In EBV-associated lymphoma, latent membrane protein 1 (LMP1) promotes AP1 and JAK-STAT signaling and promotes PD-L1 expression via an AP-1-dependent enhancer[12]; blockade of PD-1/PD-L1 interactions successfully inhibited EBV-induced lymphoma growth in a mouse model[13].

Expression of PD-L1 on tumor cells has been reported in patients with NKTCL[9], however, the role of PD-1/PD-L1 in the pathogenesis of NKTCL remains poorly understood.

There is a close correlation between Epstein–Barr virus (EBV) infection, PD-L1 expression and NKTCL. Almost all cases of NKTCL exhibited a positive result of EBV-encoded RNA (EBER) in situ hybridization in tumor samples. Additionally, pre- and post-treatment levels of circulating EBV DNA had significant prognostic implications for NKTCL patients[14-17].

NKTCL cells express programmed death protein ligand 1 (PDL1) and the EBV latent membrane protein 1 (LMP1) upregulates PDL1 expression through the MAPK/NF-κB pathway. Ligation of PD1 on effector T-cells with PDL1 on lymphoma cells leads to inhibition of T-cell activity, providing a potential mechanism for NK/T-cell lymphoma cells to evade immunosurveillance.

Pembrolizumab, a humanized anti-PD-1 monoclonal antibody, has revealed to be highly effective for relapsed/refractory ENKL failing L-asparaginase-containing therapy. In a recent study seven patients with relapsed NK/T-cell lymphoma failing previous L-asparaginase regimens (N=7) and allogeneic haematopoietic stem cell transplantation (HSCT) (N=2) were treated with the anti-PD1 antibody pembrolizumab and all seven patients responded, with five complete and two partial responses. In the partial responses, weaker staining for PDL1 expression was found in ~20% of cells in one of patients[18]. Nivolumab, another fully humanized anti-PD-1 monoclonal antibody, has later proven to be effective in ENKL. Three relapsed ENKL patients were treated with 80 to 360 mg of nivolumab. Two attained complete response, and the remaining one a partial response[19]. These findings suggest that checkpoint inhibitors are strongly promising for the treatment of ENKL.

Sintilimab, another fully humanized anti-PD-1 antibody with increased affinity to PD-1 than that of pembrolizumab, is implicated in the treatment of NKTCL, HL and solid tumors[20].

Our aim is to conduct a clinical trial to evaluate the activity and tolerability of the anti PD1 agent sintilimab in combination with pegaspargase for the initial treatment of previously untreated patients with advanced stage NKTCL.

This is a preparatory study for a larger future randomized study.

4. STUDY OBJECTIVES

The aim of the study is to test the hypothesis that the combination of sintilimab and pegaspargase might be an active and safe therapeutic option in patient with advanced stage NKTCL.

4.1 Primary Objective

The primary objective is to test the efficacy of sintilimab and pegaspargase in patients with advanced stage NKTCL.

4.2 Secondary Objectives

To further explore the efficacy and safety of sintilimab and pegaspargase as initial treatment of patients with advanced stage NKTCL.

5 STUDY OUTCOMES

5.1 Primary Outcome

The primary objective will be calculated using CRR at the end of cycle 6 (defined according to 2014 Lugano criteria and 2016 Refinement of the Lugano Classification lymphoma response criteria in the era of immunomodulatory therapy)

5.2 Secondary Outcomes

- ORR at the end of cycle 6 (defined according to 2014 Lugano criteria and 2016 Refinement of the Lugano Classification lymphoma response criteria in the era of immunomodulatory therapy)
- 2-year progression free survival
- 2-year overall survival
- Duration of response
- EBV-DNA load change
- Rate of adverse events coded according to CTCAE ver 4.0
- QoL
- Treatment-related mortality

6. STUDY DESIGN AND STUDY POPULATION

6.1 General Study Design

This phase II, open-label, single arm trial will evaluate the efficacy and safety of anti-PD-1 antibody in combination with pegaspargase in treatment of newly diagnosed advanced stage NK/T-cell lymphoma.

All eligible patients will receive induction treatment for six cycles of Anti-PD-1 antibody sintilimab plus pegaspargase (21-day cycle) and sintilimab monotherapy maintenance treatment for about 2 years (21-day cycle).

The follow-up period will last up to 2 years from the date of last subject enrollment.

Response assessment
Response will be evaluated according to 2014 Lugano criteria and 2016 Refinement of the Lugano Classification lymphoma response criteria in the era of immunomodulatory therapy using CT scan and PET-CT scan.

Patients will be evaluated for safety and tolerability of study treatment.
Anti-tumor activity assessment will be performed at cycle 3 and 6, every 4 months in the first year of follow-up, followed by every 6 months in the second year of follow-up (end of study).
For patients who experience PD or who discontinue the study due to an adverse event (AE) or a serious adverse event (SAE), the full assessment (EOT) should be obtained 30 days of the last dose of study treatment or before another anticancer therapy is initiated, whichever is shorter.

Figure 1– Study Flowchart

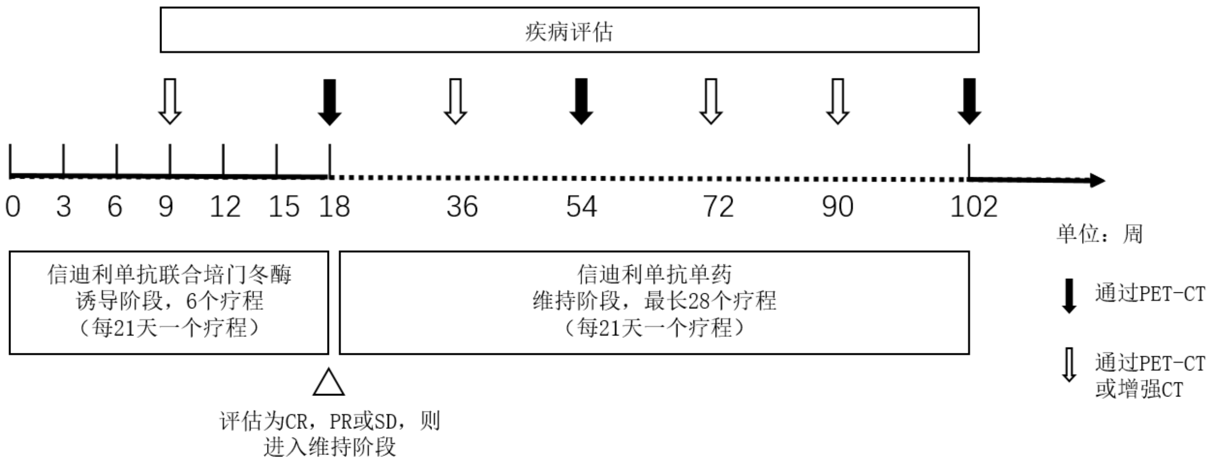

6.2 Eligibility Criteria

6.2.1 Inclusion Criteria

To be eligible for inclusion, each patient must fulfil all of the following criteria:

1. Coonfirmed histological diagnosis of NKTCL nasal type
2. No previous anti-lymphoma treatment
3. Age > 18 years
4. Ann Arbor stage III-IV
5. At least one measurable/evaluable site after diagnostic biopsy before treatment start
6. ECOG performance status of 0-2
7. Adequate hematological and organ function; i.e.
   ANC >1000 cells /mmc, platelet counts > 50.000/mmc, Hemogolbin > 9 g/dl
   AST, ALT <3 x ULN; serum bilitubin < 1.5x ULN (patient with Gilbert diasease can be enrolled)
   Serum creatinine < 2 x ULN or creatinine clearance > 50ml/min
8. Tumor tissue (fresh preferred, archivial tissue is also acceptable)
9. For women of childeberaing potential a negative pregnancy test on day 1 of cycle 1 and agree to adopta adequate measure to avoid pregnancy during study treatment and for at least one year from EOT.
10. For men agreement to remain abstinent or to use barrier contraception
11. Signed Informed consent

6.2.2 Exclusion Criteria

Patients who fulfill any of the following criteria will be excluded:

1. Confirmed histological diagnosis of aggressive NK cell leukemia
2. Early stage disease (AA stage I-II)
3. Evidence of suspect of CNS disease.
4. Has an active autoimmune disease that has required systemic treatment in past 2-years (ie, with use of disease modifying agents, corticosteroids or immunosuppressive drugs), including but not limited to myatenia gravis, myositis, autoimmune hepatitis, systemic lupus erythematosus, rheumatoid arthritis, inflammatory bowel disease, vascular thrombosis associate with antiphospholipid syndrome, wegener's granulomatosis, Sjogren syndrome, guillan barreè syndrome, multiple sclerosis, vasculitis or glomerulonephritis. The following exception are allowed: patients with autoimmune related hyopthiroidism or type I diabetes mellitus who are on stable treatment. Replacement therapy (eg, thyroxine, insulin, or physiologic corticosteroid replacement therapy for adrenal or pituitary insufficiency) is not considered a form of systemic treatment and is allowed.
5. Treatment with systemic immunosuppressive medications, including prednisone, cyclophosphamide, azathioprine, methotrexate, thalidomide and anti tumor necrosis factor (anti-TNF) agents within 2 weeks prior to cycle 1 day 1; inhaled corticosteroids are allowed.

6. Active infection requiring systemic therapy
7. History of (non-infectious) pneumonitis that required steroids; evidence of interstitial lung disease or active, non-infectious pneumonitis
8. Significant cardiovascular disease, myocardial infarciotn in the previous 3 months, unstable arrhythmias, or unstable angina.
9. History of other(s) inflitrating cancer(s) in the previous 3 years that were not treated with curative intent or who are still receiving anticancer therapy (including hormone therapy for breast or prostate cancer).
10. HBsAg, HCV or HIV positivity. Positive serology is admitted for HBV and HCV but DNA/RNA test must be negative
11. Prior therapy with an anti-PD-1, anti-PD-L1, or anti-PD-L2 agent
12. Pregnant or lactating women
13. Administration of a live attenuated vaccine within 4 weeks before cyle 1 day 1. Patients must not receive live, attenuate vaccines, including influenza vaccines at any time during study.
14. Other uncontrollable medical condition that may that may interfere the participation of the study

6.2.3 Criteria for Withdrawal / Discontinuation of Participants

Patients might have continued with therapy unless any of the following occurs:

1. Progressive disease;
2. Unacceptable toxicity dictating cessation of treatment;
3. Any change in medical status of the patient (including pregnancy) such that the Investigator believed that patient safety was compromised or that it was in the best interest of the patient to stop treatment;
4. Withdrawal of consent (included patients deciding to discontinue completely his/her study participation for any reason and refusing the Sponsor any authorization to collect information about his/her disease status: in this case no further evaluation was to be performed and no attempts were to be made to collect additional data);
5. Patient’s refusal to continue the study treatment (included patients deciding to discontinue completely his/her study participation for any reason but accepting to be followed up by the Sponsor for the collection of information regarding his/her disease status);
6. Non-compliance by the patient with protocol requirements;
7. Patient lost to follow-up. If a patient did not return for scheduled visits, every effort was to be made to re-establish contact. In any circumstance, every effort was to be made to document patient outcome;

7. STUDY INTERVENTION

7.1 Induction phase

Pegaspargase 2500IU/ m2 administered by intramuscular injection on Day 1 of each 21-day cycle for 6 cycles.
Sintilimab 200mg administered intravenously on Day 2 of each 21-day cycle for 6 cycles.

7.2 Maintenance phase

Sintilimab 200mg administered intravenously on Day 1 of each 21-day cycle for up to 28 cycles in maintenance treatment.

7.3 Prophylaxis for CNS relapse and Radiation

Patients with involvement of bone marrow, nasal or paranasal sinuses, orbit, breast, kidney, adrenal gland, or testis received prophylactic intrathecal methotrexate 10mg and cytarabine 50mg for 4 cycles during induction treatment.

For patients with limited lesions or residual disease at the end of induction treatment, radiation is recommended upon Investigators' consideration.

7.4 Instructions for Initiation of a New Cycle

A new course of treatment will begin on the scheduled Day 1 of a new cycle if:

1. ANC >1000 cells /mmc
2. platelet counts > 50.000/mmc
3. irAEs resolved to Grade 0 or 1

7.5 Management and monitoring of AEs

Dose modifications: there will be no dose modifications in this study. Patients with severe chemotherapy toxicity or patients who delay chemotherapy for more than 2 weeks due to adverse events are recommended to withdraw from the study.

Sintilimab treatment may be temporarily suspended in patients experiencing toxicity considered related to study treatment. If corticosteroids are initiated for treatment of the toxicity, the must be taperd over > 1 month to <10 mg/day or oral prednisone or equivalent before sintilimab can be resumed. If sintilimab is withheld for > 12 weeks treatment can be resumed prior approval by the principal investigator. In Table 1 is reported a guideline for the management of patients who experienced specific adverse events (see also Appendix 5).

AEs associated with sintilimab exposure may represent an immunologic etiology. These immune-related AEs (irAEs) may occur shortly after the first dose or several months after the last dose of sintilimab treatment and may affect more than on body system simultaneously. Therefore, early recognition and initiation of treatment is critical to reduce complications. Based on existing clinical study data, most irAEs were reversible and could be managed with interruptions of sintilimab, administration

of corticosteroids and/or other supportive care. For suspected irAEs, ensure adequate evaluation to confirm etiology or exclude other causes. Additional procedures or tests such as bronchoscopy, endoscopy, skin biopsy may be included as part of the evaluation. Based on the severity of irAEs, withhold or permanently discontinue sintilimab and administer corticosteroids. Dose modification and toxicity management guidelines for irAEs associated with sintilimab are provided in Table 1.

Table 1: Recommended treatment modifications for Sintilimab

Immune-related AEs	Toxicity grade or conditions (CTCAEv4.0)	Action taken to Sintilimab	irAE management with corticosteroid and/or other therapies	Monitor and follow-up
Pneumonitis	Grade 2	Withhold	• Administer corticosteroids (initial dose of 1-2 mg/kg prednisone or equivalent) followed by taper	• Monitor participants for signs and symptoms of pneumonitis<br>• Evaluate participants with suspected pneumonitis with radiographic imaging and initiate corticosteroid treatment<br>• Add prophylactic antibiotics for opportunistic infections
	Grade 3 or 4, or recurrent Grade 2	Permanently discontinue		
Diarrhea / Colitis	Grade 2 or 3	Withhold	• Administer corticosteroids (initial dose of 1-2 mg/kg prednisone or equivalent) followed by taper	• Monitor participants for signs and symptoms of enterocolitis (ie, diarrhea, abdominal pain, blood or mucus in stool with or without fever) and of bowel perforation (ie, peritoneal signs and ileus).<br>• Participants with ≥ Grade 2 diarrhea suspecting colitis should consider GI consultation and performing endoscopy to rule out colitis.<br>• Participants with diarrhea/colitis should be advised to drink liberal quantities of clear fluids. If sufficient oral fluid intake is not feasible, fluid and electrolytes should be substituted via IV infusion.
	Grade 4	Permanently discontinue		
AST / ALT elevation or Increased bilirubin	Grade 2	Withhold	• Administer corticosteroids (initial dose of 0.5- 1 mg/kg prednisone or equivalent) followed by taper	• Monitor with liver function tests (consider weekly or more frequently until liver enzyme value returned to baseline or is stable
	Grade 3 or 4	Permanently discontinue	• Administer corticosteroids (initial dose of 1-2 mg/kg prednisone or equivalent) followed by taper	
Type 1 diabetes mellitus (T1DM) or Hyperglycemia	Newly onset T1DM or Grade 3 or 4 hyperglycemia associated with evidence of β-cell failure	Withhold	• Initiate insulin replacement therapy for participants with T1DM<br>• Administer anti-hyperglycemic in participants with hyperglycemia	• Monitor participants for hyperglycemia or other signs and symptoms of diabetes.
Hypophysitis	Grade 2	Withhold	• Administer corticosteroids and initiate hormonal replacements as clinically indicated.	• Monitor for signs and symptoms of hypophysitis (including hypopituitarism and adrenal insufficiency)
	Grade 3 or 4	Withhold or permanently discontinue[1]		
Hyperthyroidism	Grade 2	Continue	• Treat with non-selective beta-blockers (eg, propranolol) or thionamides as appropriate	• Monitor for signs and symptoms of thyroid disorders.
	Grade 3 or 4	Withhold or permanently discontinue[1]		
Hypothyroidism	Grade 2-4	Continue	• Initiate thyroid replacement hormones (eg, levothyroxine or liothyroinine) per standard of care	• Monitor for signs and symptoms of thyroid disorders.
Nephritis and Renal dysfunction	Grade 2	Withhold	• Administer corticosteroids (prednisone 1-2 mg/kg or equivalent) followed by taper.	• Monitor changes of renal function
	Grade 3 or 4	Permanently discontinue		

Myocarditis	Grade 1 or 2	Withhold	• Based on severity of AE administer corticosteroids	• Ensure adequate evaluation to confirm etiology and/or exclude other causes
	Grade 3 or 4	Permanently discontinue		
All other immune-related AEs	Intolerable/ persistent Grade 2	Withhold	• Based on type and severity of AE administer corticosteroids	• Ensure adequate evaluation to confirm etiology and/or exclude other causes
	Grade 3	Withhold or discontinue based on the type of event. Events that require discontinuation include and not limited to: Gullain-Barre Syndrome, encephalitis		
	Grade 4 or recurrent Grade 3	Permanently discontinue		

1. Withhold or permanently discontinue sintilimab is at the discretion of the investigator or treating physician.

NOTE:

For participants with Grade 3 or 4 immune-related endocrinopathy where withhold of sintilimab is required, sintilimab may be resumed when AE resolves to ≤ Grade 2 and is controlled with hormonal replacement therapy or achieved metabolic control (in case of T1DM).

Infusion related reactions

Severe infusion-related reactions, including hypersensitivity and anaphylaxis, have been reported in patients receiving sintilimab. For severe infusion reactions, infusion should be stopped and sintilimab permanently discontinued (see Table 2). Patients with mild or moderate infusion reaction may continue to receive sintilimab with close monitoring; premedication with antipyretic and antihistamine may be considered.

Table 2 Sintilimab Infusion Reaction Dose modification and Treatment Guidelines

NCI CTCAE Grade	Treatment	Premedication at Subsequent Dosing
**Grade 1**<br>Mild reaction; infusion interruption not indicated; intervention not indicated	Increase monitoring of vital signs as medically indicated until the participant is deemed medically stable in the opinion of the investigator.	None
**Grade 2**<br>Requires therapy or infusion interruption but responds promptly to symptomatic treatment (e.g., antihistamines, NSAIDs, narcotics, IV fluids); prophylactic medications indicated for ≤24 hrs	**Stop Infusion.**<br>Additional appropriate medical therapy may include but is not limited to:<br>IV fluids<br>Antihistamines<br>NSAIDs<br>Acetaminophen<br>Narcotics<br>Increase monitoring of vital signs as medically indicated until the participant is deemed medically stable in the opinion of the investigator.<br>If symptoms resolve within 1 hour of stopping drug infusion, the infusion may be restarted at 50% of the original infusion rate (e.g. from 100 mL/hr to 50 mL/hr). Otherwise dosing will be held until symptoms resolve and the participant should be premedicated for the next scheduled dose.<br>**Participants who develop Grade 2 toxicity despite adequate premedication should be permanently discontinued from further study drug treatment**	Participant may be premedicated 1.5h (± 30 minutes) prior to infusion of _____ with:<br>Diphenhydramine 50 mg po (or equivalent dose of antihistamine).<br>Acetaminophen 500-1000 mg po (or equivalent dose of analgesic).
**Grades 3 or 4**<br>Grade 3:<br>Prolonged (i.e., not rapidly responsive to symptomatic medication and/or brief interruption of infusion); recurrence of symptoms following initial improvement; hospitalization indicated for other clinical sequelae (e.g., renal impairment, pulmonary infiltrates)<br>Grade 4:<br>Life-threatening; pressor or ventilatory support indicated	**Stop Infusion.**<br>Additional appropriate medical therapy may include but is not limited to:<br>Epinephrine**<br>IV fluids<br>Antihistamines<br>NSAIDs<br>Acetaminophen<br>Narcotics<br>Oxygen<br>Pressors<br>Corticosteroids<br>Increase monitoring of vital signs as medically indicated until the participant is deemed medically stable in the opinion of the investigator.<br>Hospitalization may be indicated.<br>**In cases of anaphylaxis, epinephrine should be used immediately.<br>**Participant is permanently discontinued from further study drug treatment.**	No subsequent dosing
Appropriate resuscitation equipment should be available at the bedside and a physician readily available during the period of drug administration.<br>For further information, please refer to the Common Terminology Criteria for Adverse Events v4.0 (CTCAE) at http://ctep.cancer.gov		

7.6 Concomitant therapy

Since sintilimab is cleared from the circulation through catabolism, no metabolic drug-drug interactions are expected. The use of systemic corticosteroids or immunosuppressants before starting sintilimab should be avoided because of their potential interference with the pharmacodynamic activity and efficacy of sintilimab. However, systemic corticosteroids or other immunosuppressants can be used after starting sintilimab to treat immune-related adverse reactions.

Permitted medications

Oral contraceptives, hormone replacement therapies, or other maintenance therapy are allowed to continue
Use of inhaled corticosteroids and mineralocorticoids is allowed
Megestrol administered as an appetite stimulant is acceptable
Prophilaxis of nausea and vomiting is allowed as perinstitutional practice
Use of antimicrobial and/or anti viral prophilaxis according to institutional guidelines is allowed. This also include the use of medications to prevent hepatitis B reactivation.
Flu vaccination should be given during flu season but live attenuated vacines must be avoided.

Prohibited medications

Patients should not receive long term treatment with corticosteroids other than intermittent corticosteroids for prophilaxis or for treatment of autoimmune conditions. Non steroidal hormones for non-lymphoma related conditions are permitted

Any other chemotherapy agent is not allowed.

Traditional herb medicine should not be administered.

Patients are not allowed to receive immunostimulatory medications, including but not limited to IFNalpha, IFN gamma or IL2.

Patients are not allowed to receive immunosuppressive medications, including but not limited to cyclophosphamide, azathioprine, methotrexate, lenalidomide and thalidomide.

8 STUDY ASSESSMENTS

8.1 Schedule of Events

Table 3 Schedule of Events and related footnotes summarize information on the timing of study assessments.

Protocol Activities	Pre-Treatment Assessment	Induction Phase		Maintenance Phase		Follow-up Phase	
		D1 (from Cycle 1 to Cycle 6)	EOI (14-21d from cycle 6)	D1 (from Cycle 7 to 34 cycles)	Evaluation (14-21d from cycle 12, 18, 24, 30)	EOT (within 30d from cycle 34)	FUP (every 4 month)
Informed Consent	X						
Medical history	X						
Bone marrow aspiration biopsies	X						
Tumor biopsies	X						
Pregnancy test (women of childbearing age)	X						
Physical examination	X	X	X	X	X	X	
ECOG	X	X	X	X	X	X	
CBC, urine, and stool analysis	X	X	X (CBC)	X	X (CBC)	X (CBC)	
DIC	X	X		X			
Liver and renal function, electrolytes, LDH, B2M, CRP, Pro-BNP	X	X	X	X	X	X	
EBV DNA	X	X	X	X	X	X	
UCG, EKG	X	X		X			
FT3, FT4, TSH	X	X		X			
HBV, HCV and HIV serology	X						
Tumor Imaging (evaluation)	X		X	X	X	X	
EORTC QLQ-C30	X		X	X	X	X	
Concomitant Medications	X	X	X	X		X	
AEs Assessment		X	X	X		X	
Survival						X	X
Pegaspargase		X					
Sintilimab		X		X			

8.2 Assessment of Tumor response

Tumor response is classified according to 2014 Lugano criteria[21] and 2016 Refinement of the Lugano Classification lymphoma response criteria in the era of immunomodulatory therapy[22]. The objective efficacy is divided into complete

remission (CR), partial remission (PR), stable (SD), disease recurrence or disease progression.

Response wil be assessed at the interim (C3) and the end (C6) of induction therapy and the every 6 cycles of sintilimab treatment until completion of maintenance phase (EOT visit).

8.3 Adverse Event Reporting Requirements

8.3.1 Assessment of AEs

Subjects will be evaluated for AEs at each visit with the NCI CTCAE v4.0.

Adverse events, SAEs, and other reportable safety events will be reported by the participant (or, when appropriate, by a caregiver, surrogate, or the participant's legally authorized representative).

The investigator and any designees are responsible for detecting, documenting, and reporting events that meet the definition of an AE or SAE as well as other reportable safety events. Investigators remain responsible for following up AEs, SAEs, and other reportable safety events for outcome.

The investigator, who is a qualified physician, will assess events that meet the definition of an AE or SAE as well as other reportable safety events with respect to seriousness, intensity/toxicity and causality.

8.3.1 Recording of AEs

All adverse events will be recorded on a patient specific adverse event log.

AE Reporting

Toxicity will be scored using CTCAE Version 4.0 for toxicity and adverse event reporting. A copy of the CTCAE Version 4.0 can be downloaded from the CTEP homepage (http://ctep.info.nih.gov). All adverse clinical experiences, whether observed by the investigator or reported by the patient, must be recorded, with details about the duration and intensity of each episode, the action taken with respect to the test drug, and the patient’s outcome. The investigator must evaluate each adverse experience for its relationship to the test drug and for its seriousness. The investigator must appraise all abnormal laboratory results for their clinical significance. If any abnormal laboratory result is considered clinically significant, the investigator must provide details about the action taken with respect to the test drug and about the patient’s outcome.

Serious Adverse Event (SAE) Reporting

Definition of SAE

A serious adverse event is one that at any dose (including overdose):

1. Results in death
2. Is life-threatening*
3. Requires inpatient hospitalization or prolongation of existing hospitalization
4. Results in persistent or significant disability or incapacity§

5. Is a congenital anomaly or birth defect
6. Is an important medical event¶

* “Life-threatening” means that the subject was at immediate risk of death at the time of the serious adverse event; it does not refer to a serious adverse event that hypothetically might have caused death if it were more severe.
§ “Persistent or significant disability or incapacity” means that there is a substantial disruption of a person’s ability to carry out normal life functions.
¶ Medical and scientific judgment should be exercised in deciding whether expedited reporting is appropriate in situations where none of the outcomes listed above occurred. Important medical events that may not be immediately life-threatening or result in death or hospitalization but may jeopardize the patient or may require intervention to prevent one of the other outcomes listed in the definition above should also usually be considered serious. Examples of such events include allergic bronchospasm requiring intensive treatment in an emergency room or at home, blood dyscrasias or convulsions that do not result in inpatient hospitalization, or the development of drug dependency or drug abuse. A new diagnosis of cancer during the course of a treatment should be considered as medically important.

Reporting of SAE to Institutional Review Board (IRB)

All SAEs occurring on this study will be reported to the IRB according to the IRB policy.

9. STATISTICAL CONSIDERATIONS

9.1 Sample Size Estimation

Sample size is defined considering CRR as primary study enpoint. Based on the publication by Yamaguchi et al. CRR for newly diagnosed NKTCL with stage III-IV was 40% for regimen of SMILE. Thus, 40% CRR from published study defines null hypothesis (H0) for our trial. The sample size of 20 pts will allow to demonstrate a 30% improvement of CRR from 40% (null hypothesis, H0) with a type 1 error rate=0.05 (two-sided) and power=0.80. Sample size will be 22 under consideration of drop off rate for 2 subjects.

9.2 Analysis Plan

The data will be analysed at the end of the study. No formal interim analyses are planned as the treatment is considered to be relatively safer than the standard therapy. However, analysis may be performed for conference presentations after all patients have been recruited and treated, but have not completed the follow-up time. This may only be done after recruitment of all patients and completion of treatment and hence unlikely to affect the study.

9.2.1 Efficacy Analysis

Analysis Populations

Main and secondary study objectives will be assessed with an intent to treat analysis. (i.e. all enrolled patients)

Overall Response Rate

Overall response rate is calculated as the sum of the complete and partial remissione rates.

Progression-Free Survival

Progression-free survival (PFS) will be computed from the date of enrollment to the date of disease progression or relapse or date of death for any reason or censored at the date of the last follow-up visit up, whichever occurs earlier.
PFS curves will be generated by applying the Kaplan-Meier method.

Survival

Time to survival will be computed from the date of enrollment to the date of death for any reason or censored at the date of the last contact, whichever occurs earlier.

9.2.2 Safety Analysis

The safety analysis will be conducted on the Intention to Treat (ITT) population.

10. REFERENCES

1. Tse E, Kwong YL. Diagnosis and management of extranodal NK/T cell lymphoma nasal type. Expert Rev Hematol. 2016;9(9):861–71.
2. Suzuki R, Suzumiya J, et al. Prognostic factors for mature natural killer (NK)-cell neoplasms: aggressive NK-cell leukemia and extranodal NK-cell lymphoma, nasal-type. Ann Oncol. 2010;21(5):1032-40.
3. Kwong YL, Kim WS, et al. SMILE for natural killer/T-cell lymphoma: analysis of safety and efficacy from the Asia Lymphoma Study Group. Blood. 2012;120:2973-80.
4. 中国临床肿瘤学会(CSCO)等. 培门冬酶治疗急性淋巴细胞白血病和恶性淋巴瘤中国专家共识. 2015; 42(24): 1149-58.
5. Yamaguchi M, Oguchi M, et al. Extranodal NK/T-cell lymphoma: Updates in biology and management strategies. Best Pract Res Clin Haematol. 2018; 31(3):315-21.
6. Yamaguchi M, Kwong YL, et al. Phase II study of SMILE chemotherapy for newly diagnosed stage IV, relapsed, or refractory extranodal natural killer(NK)/T-cell lymphoma, nasal type: the NK-cell tumor study group study. J Clin Oncol. 2011;29:4410-6.
7. Ahmadzadeh, M., et al., Tumor antigen-specific CD8 T cells infiltrating the tumor express high levels of PD-1 and are functionally impaired. Blood, 2009. 114(8): p. 1537-44.
8. Keir, M.E., et al., PD-1 and its ligands in tolerance and immunity. Annu Rev Immunol, 2008. 26: p. 677-704.
9. Chen, B.J., et al., PD-L1 expression is characteristic of a subset of aggressive B-cell lymphomas and virus-associated malignancies. Clin Cancer Res, 2013. 19(13): p. 3462-73.
10. Rossille, D., et al., High level of soluble programmed cell death ligand 1 in blood impacts overall survival in aggressive diffuse large B-Cell lymphoma: results from a French multicenter clinical trial. Leukemia, 2014. 28(12): p. 2367-75.
11. Green, M.R., et al., Integrative analysis reveals selective 9p24.1 amplification, increased PD-1 ligand expression, and further induction via JAK2 in nodular sclerosing Hodgkin lymphoma and primary mediastinal large B-cell lymphoma. Blood, 2010. 116(17): p. 3268-77.
12. Pianko, M.J., A.J. Moskowitz, and A.M. Lesokhin, Immunotherapy of Lymphoma and Myeloma: Facts and Hopes. Clin Cancer Res, 2018. 24(5): p. 1002-1010.
13. Ma, S.D., et al., PD-1/CTLA-4 Blockade Inhibits Epstein-Barr Virus-Induced Lymphoma Growth in a Cord Blood Humanized-Mouse Model. PLoS Pathog, 2016. 12(5): p. e1005642.
14. Lim, S.H., et al., Prognostic relevance of pretransplant Deauville score on PET-CT and presence of EBV DNA in patients who underwent autologous stem cell transplantation for ENKTL. Bone Marrow Transplant, 2016. 51(6): p. 807-12.

15. Kwong, Y.L., et al., Quantification of circulating Epstein-Barr virus DNA in NK/T-cell lymphoma treated with the SMILE protocol: diagnostic and prognostic significance. Leukemia, 2014. 28(4): p. 865-70.
16. Kim, H.S., et al., Whole blood Epstein-Barr virus DNA load as a diagnostic and prognostic surrogate: extranodal natural killer/T-cell lymphoma. Leuk Lymphoma, 2009. 50(5): p. 757-63.
17. Lei, K.I., et al., Diagnostic and prognostic implications of circulating cell-free Epstein-Barr virus DNA in natural killer/T-cell lymphoma. Clin Cancer Res, 2002. 8(1): p. 29-34.
18. Kwong, Y.L., et al., PD1 blockade with pembrolizumab is highly effective in relapsed or refractory NK/T-cell lymphoma failing l-asparaginase. Blood, 2017. 129(17): p. 2437-2442.
19. Chan TSY, Li J, et al. PD1 blockade with low-dose nivolumab in NK/T cell lymphoma failing l-asparaginase: efficacy and safety. Ann Hematol. 2018;97(1):193-196.
20. 信迪利单抗研究者手册.2017 年 11 月 28 号. V3.0.
21. Cheson BD, Fisher RI, et al. Recommendations for initial evaluation, staging, and response assessment of Hodgkin and non-Hodgkin lymphoma: the Lugano classification. J Clin Oncol. 2014;32(27):3059-68.
22. Cheson BD，Anesell S, et al. Refinement of the Lugano Classification lymphoma resoponse criteria in the era of immunomodulatory therapy. Blood. 2016;128(21): 2489-2496

APPENDIX 1 - REVISED CRITERIA FOR RESPONSE ASSESSMENT (2014)

Response and site	PET-CT based response	CT-Based response
**Complete**	**Complete metabolic response**	**Complete response (all of the following)**
Lymph nodes and extralymphatic sites	Score 1, 2, or 3* with or without a residual mass on 5PS† It is recognized that in Waldeyer's ring or extranodal sites with high physiologic uptake or with activation within spleen or marrow (e.g., with chemotherapy or myeloid colony-stimulating factors), uptake may be greater than normal mediastinum and/or liver. In this circumstance, complete metabolic response may be inferred if uptake at sites of initial involvement is no greater than surrounding normal tissue even if the tissue has high physiologic uptake.	Target nodes/nodal masses must regress to ≤ 1.5 cm in LDI. No extralymphatic sites of disease.
Non measured lesions	Not applicable	Absent
Organ enlargement	Not applicable	Regress to normal
New lesions	None	None
Bone marrow	No evidence of FDG-avid disease in marrow	Normal by morphology; if indeterminate, IHC negative
**Partial**	**Partial metabolic response**	**Partial remission (all of the following)**
Lymph nodes and extralymphatic sites	Score 4 or 5 † with reduced uptake compared with baseline and residual mass(es) of any size. At interim, these findings suggest responding disease. At end of treatment, these findings indicate residual disease.	≥50% decrease in SPD of up to 6 target measurable nodes and extranodal sites When a lesion is too small to measure on CT, assign 5 mm x 5 mm as the default value. When no longer visible, 0 x 0 mm For a node > 5 mm x 5 mm, but smaller than normal, use actual measurement for calculation.
Non measured lesions	Not applicable	Absent/normal, regressed, but no increase
Organ enlargement	Not applicable	Spleen must have regressed by ≥50% in length beyond normal.
New lesions	None	None
Bone marrow	Residual uptake higher than uptake in normal marrow but reduced compared with baseline (diffuse uptake compatible with reactive changes from chemotherapy allowed). If there are persistent focal changes in the marrow in the context of a nodal response, consideration should be given to further evaluation with MRI or biopsy or an interval scan	Not applicable
**No response or stable disease**	**No metabolic response**	**Stable disease**
Target nodes/nodal masses, extranodal lesions	Score 4 or 5 with no significant change in FDG uptake from baseline at interim or end of treatment	>50% decrease from baseline in SPD of up to 6 dominant, measurable nodes and extranodal sites; no criteria for progressive disease are met

Nonmeasured lesions<br><br>Organ enlargement<br><br>New lesions<br>Bone marrow	Not applicable<br><br>Not applicable<br><br>None<br>No change from baseline	No increase consistent with progression<br>No increase consistent with progression<br>None<br>Not applicable
**Progressive disease**	**Progressive metabolic disease**	**Progressive disease requires at least 1 of the following**
Individual target nodes/nodal masses	Score 4 or 5 with an increase in intensity of uptake from baseline and/or	PPD progression
Extranodal lesions	New FDG-avid foci consistent with lymphoma at interim or end-of-treatment assessment	An individual node/lesion must be abnormal with: LDi>1.5 cm and<br>Increase by≥50% from PPD nadir and<br>An increase in LDi or SDi from nadir<br>0.5 cm for lesions ≤ 2 cm<br>1.0 cm for lesions>2 cm<br>In the setting of splenomegaly, the splenic length must increase by>50% of the extent of its prior increase beyond baseline (e.g., a 15-cm spleen must increase to >16 cm). If no prior splenomegaly, must increase by at least 2 cm from baseline<br>New or recurrent splenomegaly
Non measured lesions	None	New or clear progression of preexisting nonmeasured lesions
New lesions	New FDG-avid foci consistent with lymphoma rather than another etiology (e.g., infection, inflammation). If uncertain regarding etiology of new lesions, biopsy or interval scan may be considered	Regrowth of previously resolved lesions<br>A new node >1.5 cm in any axis<br>A new extranodal site>1.0 cm in any axis; if <1.0 cm in any axis, its presence must be unequivocal and must be attributable to lymphoma<br>Assessable disease of any size unequivocally attributable to lymphoma
Bone marrow	New or recurrent FDG-avid foci	New or recurrent involvement

Abbreviations: 5PS, 5-point scale; CT, computed tomography; FDG, fluorodeoxyglucose; IHC, immunohistochemistry; LDi, longest transverse diameter of a lesion; MRI, magnetic resonance imaging; PET, positron emission tomography; PPD, cross product of the LDi and perpendicular diameter; SDi, shortest axis perpendicular to the LDi; SPD, sum of the product of the perpendicular diameters for multiple lesions.

*A score of 3 in many patients indicates a good prognosis with standard treatment, especially if at the time of an interim scan. However, in trials involving PET where de-escalation is investigated, it may be preferable to consider a score of 3 as inadequate response (to avoid undertreatment). Measured dominant lesions: Up to six of the largest dominant nodes, nodal masses, and extranodal lesions selected to be clearly measurable in two diameters. Nodes should preferably be from disparate regions of the body and should include, where applicable, mediastinal and retroperitoneal areas. Non-nodal lesions include those in solid organs (e.g., liver, spleen, kidneys and lungs), GI involvement, cutaneous lesions, or those noted on palpation. Non measured lesions: Any disease not selected as measured, dominant disease and truly assessable disease should be considered not measured. These sites include any nodes, nodal masses, and extranodal sites not selected as dominant or measurable or that do not meet the requirements for measurability but are still considered abnormal, as well as truly assessable disease, which is any site of suspected disease that would be difficult to follow quantitatively with measurement, including pleural effusions, ascites, bone lesions, leptomeningeal disease, abdominal masses, and other lesions that cannot be confirmed and followed by imaging. In Waldeyer's ring or in extranodal sites (e.g., GI tract, liver, bone marrow), FDG uptake may be greater than in the mediastinum with complete metabolic response, but should be no higher than surrounding normal physiologic uptake (e.g., with marrow activation as a result of chemotherapy or myeloid growth factors).
†PET 5PS: 1, no uptake above background; 2, uptake ≤ >mediastinum; 3, uptake > mediastinum but ≤liver; 4, uptake moderately >liver; 5, uptake markedly higher than liver and/or new lesions; X, new areas of uptake unlikely to be related to lymphoma.

APPENDIX 2 - RESOPONSE CRITERIA IN THE ERA OF IMMUNOMODULATORY THERAPY (2016)

Criteria	CR	PR	PD
RECIST 1.1	Disappearance of all target lesions. Any pathological lymph nodes (whether target or nontarget) must have reduction in short axis to <10 mm	At least a 30% decrease in the sum of diameters of target lesions, taking as reference the baseline sum diameters	At least a 20% increase in the sum of diameters of target lesions, taking as reference the smallest sum on study (this includes the baseline sum if that is the smallest on study). In addition to the relative increase of 20%, the sum must also demonstrate an absolute increase of at least 5 mm<br>Note: the appearance of one or more new lesions is also considered progression.
irRC	Disappearance of all lesions in two consecutive observations not less than 4 weeks apart	≥50% decrease in tumor burden compared with baseline in 2 observations at least 4 weeks apart (as measured bidimensionally)	≥25% increase in tumor burden compared with nadir (at any single time point) in 2 consecutive observations at least 4 weeks apart, where Tumor Burden = SPD index lesions + SPD new, measurable lesions
Lugano	PET-CT, score 1, 2, or 3* with or without a residual mass on 5PS† OR on CT, target nodes/nodal masses must regress to ≤1.5 cm in LDi	PET-CT score 4 or 5 with reduced uptake compared with baseline and residual mass(es) of any size. OR On CT ≥50% decrease in SPD of up to 6 target measurable nodes and extranodal sites	PET-CT score 4 or 5 with an increase in intensity of uptake from baseline and/or new FDG-avid foci consistent with lymphoma at interim or end-of-treatment assessment. OR On CT, an individual node/lesion must be abnormal with: LDi >1.5 cm and increase by ≥50% from PPD nadir and an increase in LDi or SDi from nadir 0.5 cm for lesions ≤2 cm 1.0 cm for lesions >2 cm<br>In the setting of splenomegaly, the splenic length must increase by >50% of the extent of its prior increase beyond baseline (eg, a 15-cm spleen must increase to >16 cm). If no prior splenomegaly, must increase by ≥2 cm from baseline. New or recurrent splenomegaly<br>New or clear progression of preexisiting nonmeasured lesions<br>Regrowth of previously resolved lesions<br>A new node >1.5 cm in any axis or a new extranodal site >1.0 cm in any axis; if <1.0 cm in any axis, its presence must be unequivocal and must be attributable to lymphoma<br>Assessable disease of any size unequivocally attributable to lymphoma<br>AND/OR new or recurrent involvement of the bone marrow
LYRIC	Same as Lugano	Same as Lugano	As with Lugano with the following exceptions:<br>IR<br>IR(1): ≥50% increase in SPD in first 12 weeks<br>IR(2): <50% increase in SPD with<br>a. New lesion(s), or<br>b. ≥50% increase in PPD of a lesion or set of lesions at any time during treatment<br>IR(3): Increase in FDG uptake without a concomitant increase in lesion size meeting criteria for PD

IR, immune response; LDi, longest diameter; PPD, product of the perpendicular diameters; SDi, short diameter; 5PS, 5-point scale.

*A score of 3 in many patients indicates a good prognosis with standard treatment, especially if at the time of an interim scan. However, in trials involving PET where de-escalation is investigated, it may be preferable to consider a score of 3 as inadequate response (to avoid undertreatment).

†PET 5PS: 1, no uptake above background; 2, uptake ≤ mediastinum; 3, uptake > mediastinum but ≤ liver; 4, uptake greater than liver; 5, uptake markedly higher than liver (2-3 times SUVmax in normal liver) and/or new lesions; X, new areas of uptake unlikely to be related to lymphoma.

APPENDIX 3 - ECOG PERFORMANCE STATUS SCALE

ECOG Scale	Performance Status
0	Fully active, able to carry out all pre-disease performance without restriction.
1	Restricted in physically strenuous activity, but ambulatory and able to carry out work of a light or sedentary nature, e.g. light house work, office work.
2	Ambulatory and capable of all selfcare, but unable to carry out any work activities. Up and about more than 50% of waking hours.
3	Capable of only limited selfcare, confined to bed or chair more than 50% of waking hours.
4	Completed disabled. Cannot carry out any selfcare. Totally confined to bed or chair.
5	Dead

ECOG = EASTERN COOPERATIVE ONCOLOGY GROUP

NOTE THAT:
ECOG 0 corresponds to Karnofsky performance status of100-90
ECOG 1 corresponds to Karnofsky performance status of 80-70
ECOG 2 corresponds to Karnofsky performance status of 60-50
ECOG 3 corresponds to Karnfosky performance status of 40-30
ECOG 4 corresponds to Karnofsky performance status of 20-10
ECOG 5 corresponds to Karnofsky performance status of 0

APPENDIX 4: ANN ARBOR STAGING

Stage I:
I: Involvement of a single lymph node region
IE: Localized involvement of a single extra-lymphatic organ or site
Stage II:
II: Involvement of 2 or more lymph node regions on the same side of the diaphragm
IIE: Localized involvement of a single associated extra-lymphatic organ or site and its regional lymph nodes with or without other lymph node regions on the same side of the diaphragm
Stage III:
III: Involvement of lymph node regions on both sides of the diaphragm
IIIE: Involvement of lymph node regions on both sides of the diaphragm accompanied by localized involvement of an extra lymphatic organ or site
IIIS: Involvement of lymph node regions on both sides of the diaphragm accompanied by involvement of the spleen
IIIS+E: Both IIIS+IIIE
Stage IV:
IV: Disseminated (multifocal) involvement of 1 or more extralymphatic sites with or without associated lymph node involvement or isolated extra lymphatic organ involvement with distant (non-regional) nodal involvement
IVE: Extra-nodal lymphoid malignancies arise in tissues separate from, but near, the major lymphatic aggregates.

Source: American Joint Committee on Cancer. Non-Hodgkin’s lymphoma. In: AJCC Staging Manual. 5th ed. Philadelphia, PA: Lippincott-Raven; 1997:289-294.

APPENDIX 5 - RECOMMENDED TREATMENT MODIFICATIONS FOR SINTILIMAB

**Immune-related adverse**	**Severity**	**Treatment modification**
Pneumonitis	Grade 2	Withhold until adverse reactions recover to Grade 0-1*
	Grade 3 or 4, or recurrent Grade 2	Permanently discontinue
Colitis	Grade 2 or 3	Withhold until adverse reactions recover to Grade 0-1*
	Grade 3 or 4, or recurrent Grade 3	Permanently discontinue
Nephritis	Grade 2 with creatinine > 1.5 to ≤ 3 times upper limit of normal (ULN)	Withhold until adverse reactions recover to Grade 0-1*
	Grade ≥ 3 with creatinine > 3 times ULN	Permanently discontinue
Endocrinopathies	Symptomatic hypophysitis<br>Type 1 diabetes associated with Grade ≥3 hyperglycaemia (glucose > 250 mg/dL or > 13.9 mmol/L) or associated with ketoacidosis.<br>Hyperthyroidism Grade ≥ 3	Withhold until adverse reactions recover to Grade 0-1* For patients with Grade 3 or Grade 4 endocrinopathy that improved to Grade 2 or lower and is controlled with hormone replacement, if indicated, continuation of sintilimab may be considered after corticosteroid taper, if needed. Otherwise treatment should be discontinued. Hypothyroidism may be managed with replacement therapy without treatment interruption.
Hepatitis	Grade 2 with aspartate aminotransferase (AST) or alanine aminotransferase (ALT) > 3 to 5 times ULN or total bilirubin > 1.5 to 3 times ULN	Withhold until adverse reactions recover to Grade 0-1*
	Grade ⩾ 3 with AST or ALT > 5 times ULN or total bilirubin > 3 times ULN	Permanently discontinue
	In case of liver metastasis with baseline Grade 2 elevation of AST or ALT, hepatitis with AST or ALT increases ⩾ 50% and lasts ⩾ 1 week	Permanently discontinue
Skin reactions	Grade 3 or suspected Stevens-Johnson syndrome (SJS) or toxic epidermal<br>necrolysis (EN)	Withhold until adverse reactions recover to Grade 0-1*
	Grade 4 or confirmed SJS or TEN	Permanently discontinue
Other immune-related adverse reactions	Based on severity and type of reaction (Grade 2 or Grade 3)<br><br>Grade 3 or 4 myocarditis<br>Grade 3 or 4 encephalitis<br>Grade 3 or 4 Guillain-Barré syndrome<br><br>Grade 4 or recurrent Grade 3	Withhold until adverse reactions recover to Grade 0-1*<br><br>Permanently discontinue<br><br><br><br>Permanently discontinue
Infusion-related reactions	Grade 3 or 4	Permanently discontinue
Note: toxicity grades are in accordance with National Cancer Institute Common Terminology Criteria for Adverse Events Version 4.0 (NCI-CTCAE v.4).		

* If treatment-related toxicity does not resolve to Grade 0-1 within 12 weeks after last dose of KEYTRUDA, or if corticosteroid dosing cannot be reduced to ≤ 10 mg prednisone or equivalent per day within 12 weeks, KEYTRUDA should be permanently discontinued.
